# Dengue subgenomic flaviviral RNA disrupts immunity in mosquito salivary glands to increase virus transmission

**DOI:** 10.1371/journal.ppat.1006535

**Published:** 2017-07-28

**Authors:** Julien Pompon, Menchie Manuel, Geok Kee Ng, Benjamin Wong, Chao Shan, Gayathri Manokaran, Ruben Soto-Acosta, Shelton S. Bradrick, Eng Eong Ooi, Dorothée Missé, Pei-Yong Shi, Mariano A. Garcia-Blanco

**Affiliations:** 1 Programme in Emerging Infectious Diseases, Duke-NUS Medical School, Singapore; 2 UMR IRD-CNRS MIVEGEC, IRD, Montpellier, France; 3 Department of Biochemistry and Molecular Biology, University of Texas Medical Branch, Galveston, TX, United States of America; University of Washington, UNITED STATES

## Abstract

Globally re-emerging dengue viruses are transmitted from human-to-human by *Aedes* mosquitoes. While viral determinants of human pathogenicity have been defined, there is a lack of knowledge of how dengue viruses influence mosquito transmission. Identification of viral determinants of transmission can help identify isolates with high epidemiological potential. Additionally, mechanistic understanding of transmission will lead to better understanding of how dengue viruses harness evolution to cycle between the two hosts. Here, we identified viral determinants of transmission and characterized mechanisms that enhance production of infectious saliva by inhibiting immunity specifically in salivary glands. Combining oral infection of *Aedes aegypti* mosquitoes and reverse genetics, we identified two 3’ UTR substitutions in epidemic isolates that increased subgenomic flaviviral RNA (sfRNA) quantity, infectious particles in salivary glands and infection rate of saliva, which represents a measure of transmission. We also demonstrated that various 3’UTR modifications similarly affect sfRNA quantity in both whole mosquitoes and human cells, suggesting a shared determinism of sfRNA quantity. Furthermore, higher relative quantity of sfRNA in salivary glands compared to midgut and carcass pointed to sfRNA function in salivary glands. We showed that the Toll innate immune response was preferentially inhibited in salivary glands by viruses with the 3’UTR substitutions associated to high epidemiological fitness and high sfRNA quantity, pointing to a mechanism for higher saliva infection rate. By determining that sfRNA is an immune suppressor in a tissue relevant to mosquito transmission, we propose that 3’UTR/sfRNA sequence evolution shapes dengue epidemiology not only by influencing human pathogenicity but also by increasing mosquito transmission, thereby revealing a viral determinant of epidemiological fitness that is shared between the two hosts.

## Author summary

Dengue is a re-emerging global disease transmitted from human-to-human by mosquitoes. While environmental and host immune factors are important, viral determinants of mosquito transmission also shape the epidemiology of dengue. Understanding how dengue viruses influence transmission will help identify isolates with high epidemic potential and untangle the evolutionary pressures at play in the dual-host cycle. Here, we identified 2 substitutions in the 3’UTR of epidemic isolates that increase transmission through immune suppression in the salivary glands. Using oral infection of *Aedes aegypti* mosquitoes, we reported that epidemic isolates produced more subgenomic flaviviral RNA (sfRNA) in salivary glands. SfRNA is generated from the 3’UTR sequence remaining after partial genome degradation by a host nuclease. Using reverse genetics, we identified the two 3’UTR substitutions responsible for the higher sfRNA quantity in salivary glands. We further showed that these substitutions increased dengue virus titer in salivary glands and rate of saliva infection, and suppressed the Toll immune response in salivary glands. Our study identifies the substitutions that determine virus epidemiological fitness and provides a mechanism for sfRNA-mediated enhancement of transmission. Together with previous work demonstrating that sfRNA sequence modification influences dengue virus pathogenicity in human, and that shows variation in sfRNA sequence when the viruses transition from one host to vector and vice versa, our study supports that sfRNA evolution is constrained in the two hosts.

## Introduction

Dengue viruses (DENV), members of the flavivirus genus, infect about 390 million people annually and threaten one third of the world population [1] making them the most important arboviruses in the world. DENV are primarily transmitted by *Aedes aegypti* mosquitoes [2]. Increase in transmission efficiency augments arbovirus epidemiological fitness (EF) [3, 4], defined hereafter as the capacity to generate an epidemic, and can trigger epidemics as observed in other mosquito-virus systems [5–7]. Successful transmission following an infectious blood meal occurs when the virus infects the midgut, escapes from this organ to multiply in secondary tissues, including hemocytes, fat bodies or muscles, and productively infects the salivary glands, from which it is expectorated along with saliva during subsequent blood feedings [3, 8, 9]. Upon infection, mosquitoes mount a powerful innate immune response [10] mediated by the Toll, IMD, Jak/STAT, RNAi, and/or TRAF/Rel2 pathways that can inhibit DENV [11–17]. Transmission efficiency is likely dictated by the balance of power between host antiviral measures and viral countermeasures [18, 19]. Identification of the viral factors that interfere with the immune response will reveal viral strategies to enhance transmission and facilitate identification of viruses with high EF.

One such factor is the subgenomic flaviviral RNA fragment (sfRNA), produced from partial degradation of flavivirus genomes by the host exonuclease XRN1 that stalls at 3’UTR nuclease resistant structures, functionally labelled XRN1-resistant RNAs (xrRNAs) [20–23]. Different species of DENV sfRNA can be produced from the 3’UTR sequence depending on which xrRNA the degradation stalls at [24]. In mosquitoes, sfRNA from DENV-1 (serotype 1) and West Nile virus (WNV) can suppress RNAi processing of an ectopic substrate by inhibiting Dicer-2 cleavage [25], likely through direct binding [26]. Nonetheless, a recent *in vivo* study demonstrated the lack of RNAi alteration by WNV sfRNA in spite of sfRNA processing by the RNAi machinery [27]. The study further showed a transmission decrease by *Culex* mosquitoes (the main vector of WNV) for sfRNA-deficient WNV, and indicated that reduction of transmission potential was mediated by an unidentified mechanism in midgut. Phylogenetic clustering of flavivirus 3’UTR sequences according to vector species suggests functional specialization for sfRNAs in different vector-virus system [28]. In humans, sfRNA alters innate immune response by altering interferon production and the cellular response to interferons [29–32]. Evolution of the DENV-2 (serotype 2) sfRNA sequence in more fit viruses increases production of this non-coding RNA and/or augments binding-specificity to innate immune regulators, both concurrently antagonizing the antiviral response [30]. The resulting increased infectivity provided an explanation for a DENV-2 lineage replacement that occurred in Puerto Rico in 1994–1995 (PR-2B replacing PR-1 isolates), which resulted in an important outbreak [33]. Altogether, these studies, and several others, suggest a strong immune suppressor function for sfRNA in both humans and mosquitoes. Nevertheless, understanding of the relationship between sfRNA evolution, its anti-immune function in mosquitoes and how these impact mosquito transmission is lacking [34]. Such knowledge would serve molecular surveillance to predict emergence of viruses with high transmission capacity and improve the characterization of the evolutionary pressures that shape sfRNA, a determinant of dengue pathogenesis[30].

To address this gap in our knowledge, we tested whether or not variant 3’UTR sequences alter sfRNA quantity in mosquitoes and investigated how these influence transmission. We made use of an existing bank of DENV-2 isolates collected in Puerto Rico with different EF and variable 3’UTR sequences [30, 33]. After oral infection of *Ae*. *aegypti* mosquitoes with high and low EF isolates, we observed that, although overall levels of viral genomes (gRNA) and progeny were not different between these, high EF isolates produced higher sfRNA:gRNA ratios. Using two representatives of each epidemic level, we noted significantly higher sfRNA:gRNA ratio in salivary glands for the high EF isolate. Remarkably, regardless of the isolate, the sfRNA:gRNA ratio was higher in salivary glands than in midgut or carcass, suggesting a heretofore unprecedented tissue-specific regulation of sfRNA accumulation. Using reverse genetics, we demonstrated that two 3’UTR substitutions in the high EF isolate were responsible for the difference in sfRNA:gRNA ratios in salivary glands. We then found that the isolate with higher EF and the chimeric virus with the corresponding 3’UTR resulted in higher infection rate of saliva. Finally, we determined that the high EF isolate disrupted the Toll immune response in salivary glands and incriminated the same two 3’UTR substitutions that alter sfRNA quantity.

## Results

### While infectivity does not vary with epidemiological fitness, epidemic isolates produce higher sfRNA:gRNA ratio

To study whether sfRNA determines EF by influencing mosquito infection, we selected DENV-2 isolates within the Asia subtype IIIb with different EF and 3’UTR sequences. The isolates were selected from those identified in a study that examined viral phylogeny over the twenty year period from 1981–2001 in Puerto Rico [33]. This study revealed the replacement of the endemic PR-1 by an epidemic PR-2B clade over the years 1994–1995, resulting in an important outbreak [33]. Full genome sequencing of viruses from the two clades refined the phylogenetic history and implicated changes in the 3’UTR in EF [30]. Three isolates from the clade PR-2B with high EF, which we defined as such because many highly related isolates were collected during the outbreak, were selected [30] (Table 1). Isolates with lower EF included five isolates from the replaced endemic PR-1 clade and PR315022, which belongs to PR-2B clade but does not have homologous isolates from the outbreak, suggesting low EF. Sequence identity between our virus stocks and the original passage 0 isolates was validated using NGS for one representative of PR-1 (PR1940) and two of PR-2B (PR6452 and PR315022) (raw sequences available on NCBI: SRX2617313-SRX26173151). The 3’UTR sequences varied between and within the clades and all PR-2B isolates possessed the three substitutions associated with immune suppression in humans (S1 Fig) [30].

**Table 1:** Description of isolates with different epidemiological fitness collected in Puerto Rico in 1994.

Clade	Isolate	Apparent relative epidemiological fitness^1^
PR-1	PR1940	Low
	PR2974	Low
	PR0013	Low
	PR4056	Low
	PR8545	Low
PR-2B	PR6452	High
	PR6913	High
	PR9963	High
	PR315022	Low

^1^Clade is determined based on a phylogeny study [33] and full genome sequencing [30]. Apparent relative EF is defined based on the clade, all PR-1 isolates were considered low EF as they were replaced by PR-2B isolates, and PR-2B isolates were defined as having high EF because of high number of homologous isolates collected during the outbreak, except for PR315022 that had none and appeared to have been a dead-end sub-lineage.

To test whether higher EF was associated with increased vector susceptibility, we orally infected *Ae*. *aegypti* mosquitoes with the aforementioned PR-1 and PR-2B isolates, and quantified feeding rate, mosquito survival and virus infectivity. To use an epidemiologically relevant inoculum dose, we offered mosquitoes an artificial blood meal containing 10^6^ pfu/ml. The inoculum concentration is in the lower range of the viremia measured in hospitalized children during the first couple of days after fever onset[35], which overlaps with the peak of transmissibility to mosquitoes[36, 37]. Mosquito feeding rate was not affected by the isolates; except for two PR1 isolates, PR2974 and PR4056, which exhibited significantly higher and lower rates, respectively (S2A and S2B Fig). Survival of mosquitoes at 10 days post-infection (p.i.) was not altered after infection with the isolates, except for PR1940, PR0013 and PR6913 that had significantly lower survival than PR315022 (S3A Fig). Importantly, survival did not segregate with EF level (S3B Fig). At 21 days p.i., survival of mosquitoes infected with three viral isolates, which represented high and low EF viruses, was not altered as compared to non-infected mosquitoes (S3C Fig). A decrease in the rate of blood feeding or in survival could diminish vector capacity and therefore virus fitness [38], but our data are not consistent with either of these parameters explaining fitness of the studied DENV-2 isolates.

**Fig 1:**
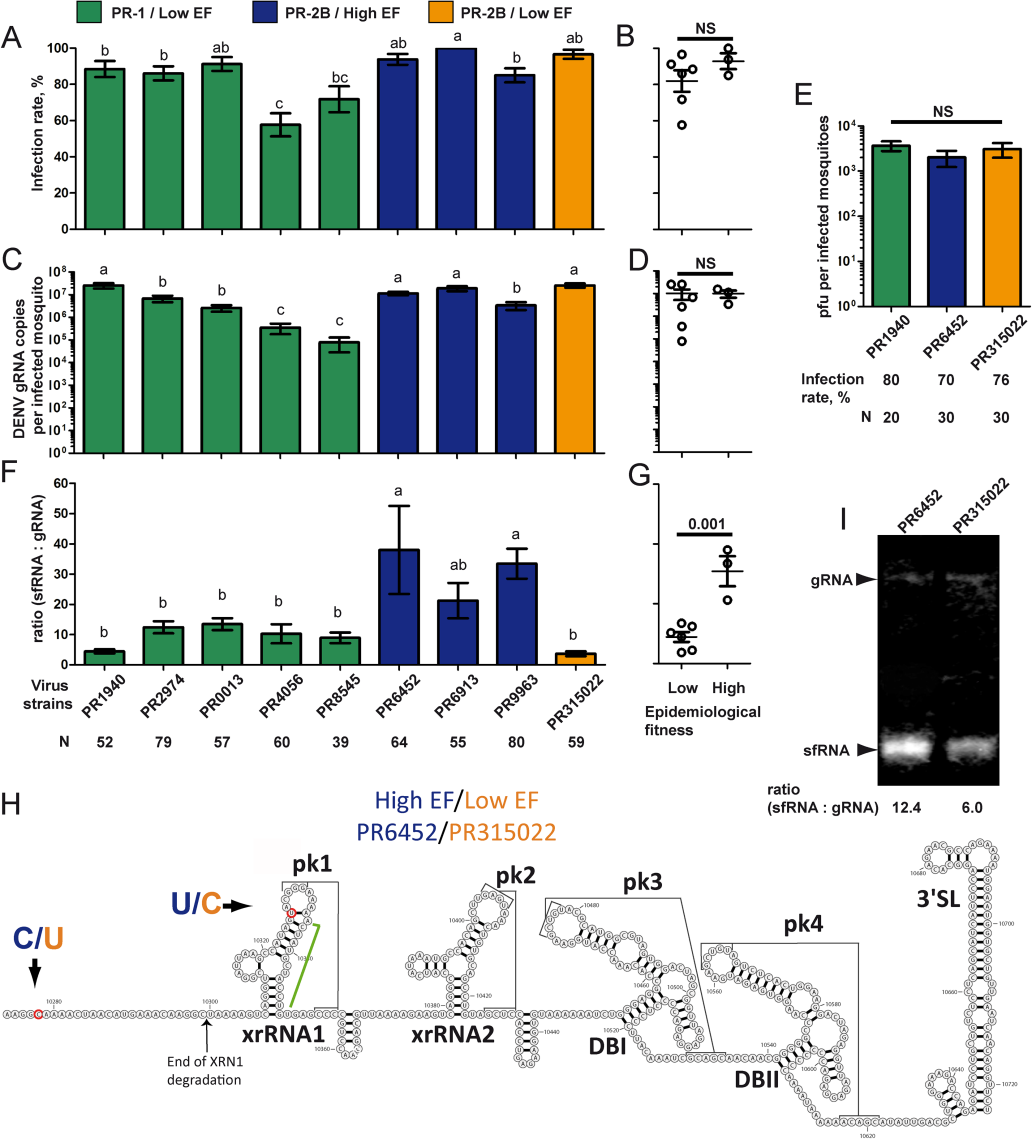
Epidemiological fitness positively correlates with higher levels of sfRNA. Mosquitoes were orally infected with 10^6^ pfu / ml of DENV-2 PR-1 or PR-2B isolates and analyzed 10 days post-infection. (A) Infection rate per isolates and (B) for the same isolates grouped by epidemiological fitness level. (C) DENV-2 gRNA copies per infected mosquito per isolates and (D) for the same isolates grouped by epidemiological fitness level. (E) Plaque forming unit (pfu) per infected mosquito. (F) Ratio of sfRNA:gRNA in whole mosquito per isolates and (G) for the same isolates grouped by epidemiological fitness level. Bars with a different letter were significantly different following Z-test (A) or Tukey’s test (C, E and F). T-test was applied to test significance for isolates grouped according to epidemiological fitness level (B, D, G). N, number of mosquitoes analyzed. Bars show percentages ± s.e. or means ± s.e.m. (H) Mapping of the 3’UTR sequence differences between PR6452 (high EF) and PR315022 (low EF). xrRNA, XRN1-nuclease resistant RNA; pk, pseudoknot; DB, dumb bell; Green line show predicted long-range nucleotide interaction based on the Zika xrRNA1 model [48]. (I) Northern blot with a 3’UTR probe on RNA extracts from whole mosquitoes 10 days p.i. with PR6452 and PR315022.

We detected and quantified DENV genomic RNA (gRNA) in whole mosquitoes 10 days p.i. using RT-qPCR (S4 Fig). Infection rate, which was defined as percentage of infected mosquitoes among blood-fed ones, varied between isolates (Fig 1A) but did not segregate with EF level (Fig 1B; p = 0.27). Similarly, the number of gRNA copies per infected mosquito varied between isolates (Fig 1C) but did not segregate with EF level (Fig 1D; p = 0.98). To determine whether the production of infectious particles was linked to EF level, we orally infected mosquitoes with the three representative isolates, PR1940, PR6452 and PR315022 (Table 1), and 10 days p.i. measured plaque forming units (PFU) from whole mosquitoes. Virus titer was not different between the three isolates (Fig 1E). Therefore, the level of viral genomes or of infectious particles in whole mosquitoes did not correlate with viral EF.

We then quantified the sfRNA copies per infected mosquito at 10 days p.i. with the same nine isolates, and calculated the ratio of sfRNA to gRNA (sfRNA:gRNA) in whole mosquitoes. The forward primer used to quantify sfRNA with RT-qPCR annealed after the xrRNA1 structure (S1 Fig) and, hence, only detects the longer sfRNA species that results from XRN1 stalling at the xrRNA1 [24]. Strikingly, the sfRNA:gRNA ratio was significantly higher for the high EF isolates than for isolates with lower EF (p = 0.001), PR-1 isolates and the PR-2B isolate, PR315022 (Fig 1F and 1G). Infection with PR315022 produced the lowest sfRNA:gRNA ratio of 3.6 (± 0.83), whereas infection with PR6452 (high EF PR-2B) resulted in the highest ratio of 38 (± 14.57). The 3’UTR sequences for PR6452 and PR315022 differ by two nucleotides: a C to U transition 5 nt downstream of the termination codon that is not expected to be in the mature sfRNA [39], and a U to C transition in the distal loop of the first nuclease resistant (xrRNA1) structure (Fig 1H). Both substitutions are absent from the other PR-1 and PR-2B isolates (S1 Fig). Substitutions in sfRNA sequences can disrupt the nuclease resistant structures and result in shorter sfRNAs [24, 39], to test whether or not this was the case between PR6452 and PR315022 we used Northern blots to compare sfRNA size and quantity in the whole body of *Ae*. *aegypti* collected 10 days p.i. with these isolates. The probe used was complementary to the 3’UTR sequence starting immediately downstream of xrRNA1 and ending at the 3’ end of the genome (S1 Fig), and can thus detect all sfRNA species [24]. The predominant sfRNA in PR315022 infected cells appeared to be very similar in size to that observed in PR6452 infected cells (Fig 1I), although high resolution Northern Blot with acrylamide gel would be required to confirm this. Further, we confirmed using Northern Blot the higher ratio of sfRNA:gRNA in PR6452-infected whole mosquitoes. Altogether, our data show an association between higher EF and higher sfRNA:gRNA ratio in mosquitoes.

### SfRNA:gRNA ratio positively correlates in human and mosquito

**Fig 2:**
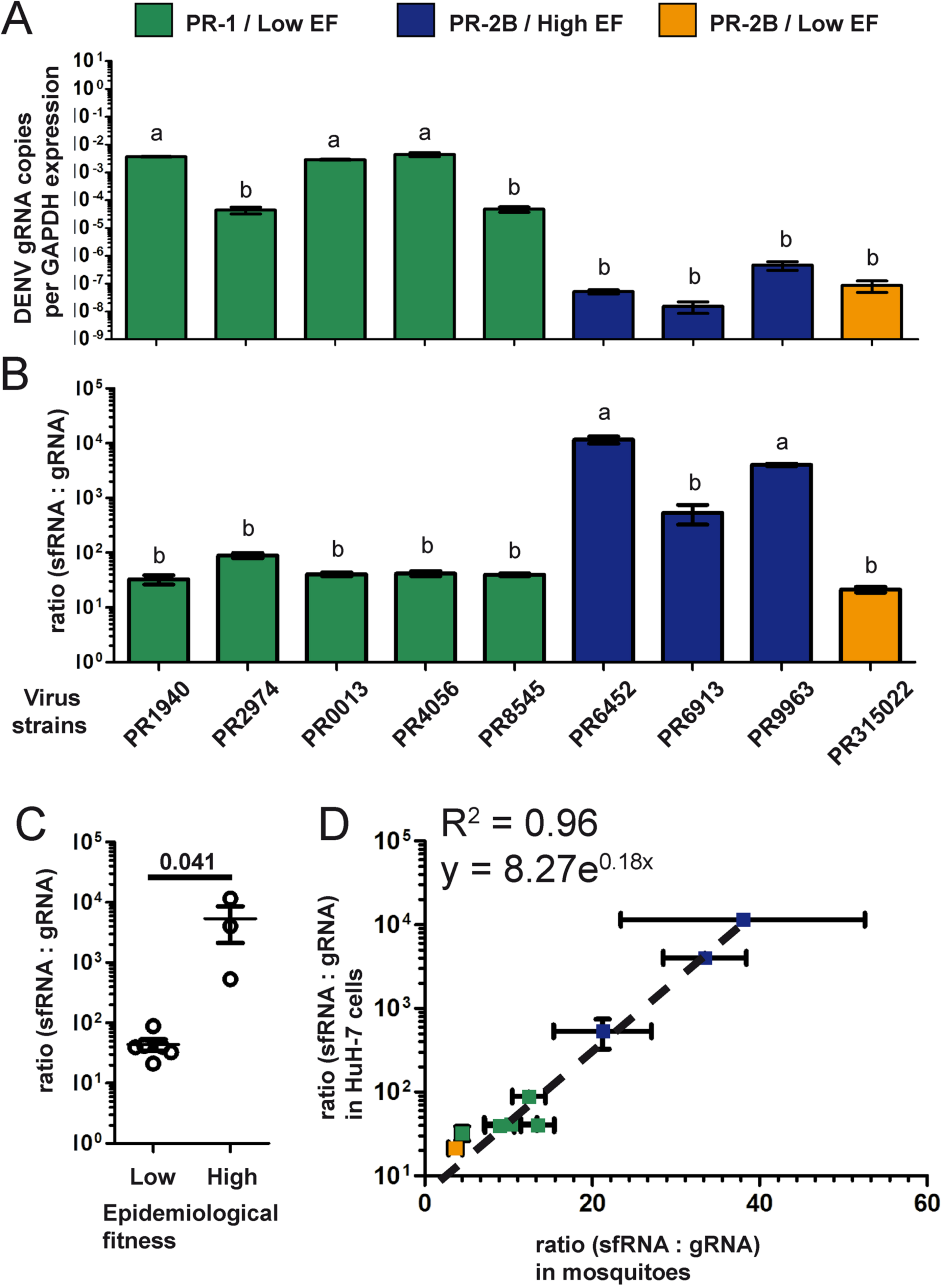
Ratios of sfRNA:gRNA in human cells and mosquitoes correlate well. HuH-7 cells were infected with the isolates. (A) gRNA copies relative to GAPDH expression and (B) sfRNA:gRNA ratio were quantified using RT-qPCR. Bars with a different letter were significantly different following Tukey’s test. (C) Correlation between sfRNA:gRNA ratios in HuH-7 cells and whole mosquitoes was plotted. The dotted line represents an exponential regression, for which the R^2^ and equation is provided within the graph. The data in HuH-7 cells have been previously used for comparing averages of PR-1 and PR-2B strains [30] but never shown for each individual strains.

To compare sfRNA production for the aforementioned nine DENV-2 isolates in human cells, we infected HuH-7 cells and quantified gRNA and sfRNA. As we have reported before [30], PR-1 isolates produced higher gRNA levels than PR-2B isolates at 24 h.p.i. (Fig 2A). However, similarly to what we report now in mosquitoes (Fig 1F and 1G), high EF isolates had higher sfRNA:gRNA ratios (Fig 2B and 2C). PR315022 was the exception among the PR-2B isolates and had a sfRNA:gRNA ratio more typical of low EF PR-1 isolates. Interestingly, the ratios in human cells and mosquitoes were positively correlated (Pearson correlation = 0.85; p = 0.004) and the best fit was an exponential regression (R^2^ = 0.96 vs. linear regression: R^2^ = 0.71) (Fig 2D), suggesting that shared determinants set sfRNA:gRNA ratios in humans and mosquitoes.

### SfRNA:gRNA ratio is higher in salivary glands and for the higher epidemiological fitness virus

**Fig 3:**
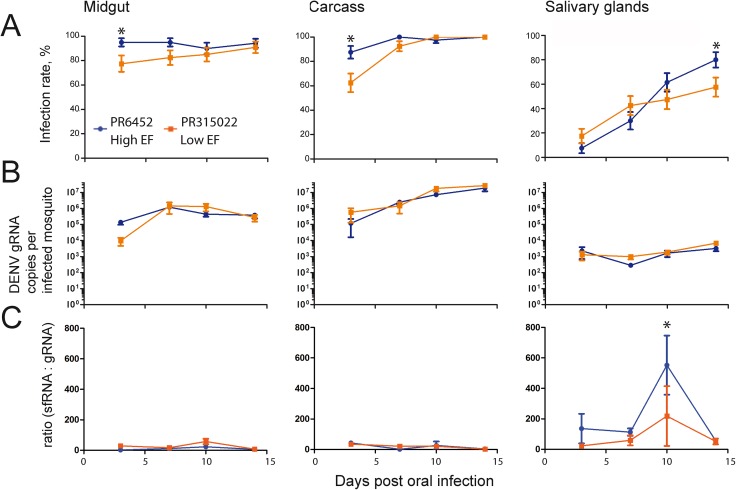
SfRNA:gRNA ratio is higher in salivary glands, peaks at 10 days post-oral infection and is higher for PR6452 than PR315022. Mosquitoes were orally challenged with 10^6^ pfu /ml of PR6452 (high EF) or PR315022 (low EF) isolates. After 3, 7, 10 and 14 days, mosquitoes were dissected into salivary glands, midgut and carcass (what is left after dissection) and the DENV gRNA and sfRNA copies were quantified. (A) Infection rate, (B) gRNA copies per infected mosquitoes and (C) sfRNA:gRNA ratio in midgut, carcass and salivary glands at different time points post-infection. Two independent experiments each with twenty mosquitoes per condition were conducted. Percentages (infection rate) ± s.e. and mean ± s.e.m (DENV gRNA copies per infected mosquitoes and sfRNA:gRNA ratio) are shown. Asterisks represent significant differences between viral isolates for the same tissue on the same day (p < 0.05).

EF for DENV depends in part on transmission efficiency. One measure of efficiency of mosquito transmission is the extrinsic incubation period, determined by the time to infect salivary glands after oral infection [3]. To test whether or not viruses with different EF have different infection kinetics, we orally infected mosquitoes with PR-2B isolates: the high EF PR6452 and the low EF PR315022. We decided to focus on these two isolates since they are both in the same PR-2B clade and yet they appear to be very different in terms of fitness. At 3, 7, 10 and 14 days p.i., we quantified DENV gRNA copies in the midgut, carcass and salivary glands. We did not pursue the experiments past 14 days p.i. as a majority of female mosquitoes do not live longer than this in wild settings [40]. Infection rate for PR6452 was significantly higher in midgut and carcass at 3 days, and in the salivary glands at 14 days p.i. Indeed, 14 days p.i., salivary gland infection rate reached 80 and 57% for PR6452 and PR315022, respectively (Fig 3A). The number of gRNA copies per infected mosquitoes was not significantly different between the two viruses in the three tissues within each time point (Fig 3B, S1 Table). The infection rate results suggest that the high EF isolate PR6452 have slightly faster kinetic of infection than the lower EF PR315022.

Quantification of sfRNA revealed two interesting observations. First, regardless of virus, sfRNA:gRNA ratio was higher in salivary glands than in midgut and carcass (Fig 3C, S2 Table). The effect of tissue was significant in our ANOVA model (S2 Table; P < 0.001) and, at 10 days p.i. which was the apparent peak of sfRNA production, there was approximately a ten-fold higher sfRNA:gRNA ratio in salivary glands than in the two other tissues. Second, the sfRNA:gRNA ratio in salivary glands was higher in PR6452 infected mosquitoes than those infected with PR315022 at 3, 7 and 10 days p.i,. and the difference was significant at 10 days p.i. Both the higher sfRNA:gRNA ratio in salivary glands and for high EF virus suggest a role for sfRNA in salivary glands in transmission efficiency.

To investigate whether or not tissue specific variations in exonuclease XRN1 expression could explain the sfRNA:gRNA ratio, we quantified *XRN1* mRNA expression in the different tissues. We orally infected mosquitoes with PR6452 or PR315022 or offered them a non-infectious blood meal, and 10 days p.i., isolated total RNA from salivary glands, midgut and carcass. *XRN1* RNA normalized to *Actin* RNA was measured by RT-qPCR. *XRN1* RNA expression was the lowest in salivary glands and was not altered by virus infection (S5A Fig). To estimate efficiency of XRN1-degradation of gRNA, we normalized *XRN1* expression to DENV gRNA. *XRN1* expression to gRNA was lower in midgut and higher in carcass than in salivary glands, and not different in mosquitoes infected with any of the isolates (S5B Fig). The results suggest that *XRN1* RNA expression could not account for differences in sfRNA:gRNA ratio between the tissues and the two isolates.

### Differences in 3’UTR sequence determine sfRNA:gRNA ratios in salivary glands

The high EF PR6452 and the lower EF PR315022 isolates have many nucleotide differences (S3 Table) that could cause the observed higher sfRNA:gRNA ratio. Since sequences in the 3’UTR are most likely to cause changes in sfRNA levels, we tested the role of the two transitions in this region on infection parameters including the sfRNA:gRNA ratio (S3 Table, Fig 1H). We constructed two chimeric viruses, based on the DENV-2 NGC isolate, identical except for the 3’UTR sequences which were derived from PR6452 or PR315022; thereafter named IC6452 and IC315022, respectively. The sequence of the two chimeric viruses were validated using NGS (raw sequences available on NCBI: SRX2617311-SRX2617312) and were as expected.

**Fig 4:**
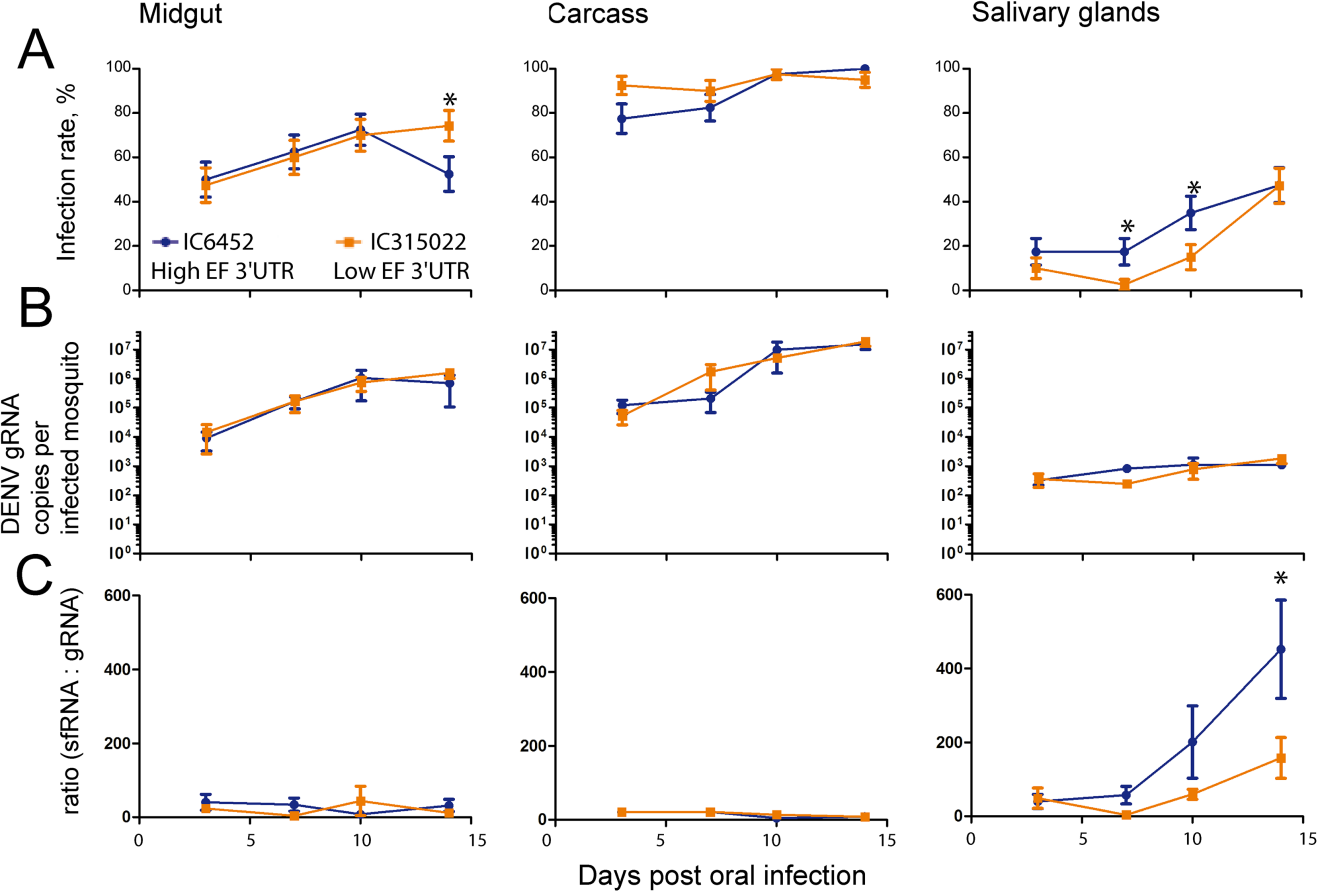
3’UTR sequences determine sfRNA:gRNA ratios in chimeric viruses and correlate with epidemiological fitness. Mosquitoes were orally challenged with 10^6^ pfu / ml of IC6452 (high EF 3’UTR) or IC315022 (low EF 3’UTR) chimeric viruses. After 3, 7, 10 and 14 days, mosquitoes were dissected into salivary glands, midgut and carcass (what is left after dissection) and the DENV gRNA and sfRNA copies were quantified. (A) Infection rate, (B) gRNA copies per infected mosquitoes and (C) sfRNA:gRNA ratio in midgut, carcass and salivary glands at different time points post-infection. Two independent experiments each with twenty mosquitoes per condition were conducted. Percentages (infection rate) ± s.e. and mean ± s.e.m (DENV gRNA copies per infected mosquitoes and sfRNA:gRNA ratio) are shown. Asterisks represent significant differences between viruses for the same tissue within the same day (p < 0.05).

The chimeric viruses were used to orally infect mosquitoes, and at 3, 7, 10 and 14 days p.i. infection rate, levels of DENV gRNA and sfRNA:gRNA ratios were quantified in midgut, carcass and salivary glands. Infection rate was not different between the two chimeric viruses in midgut and carcass at 3, 7 and 10 days p.i., but was significantly higher for IC6452 than for IC315022 in salivary glands at 7 and 10 days p.i. (Fig 4A). Salivary gland infection rate reached 17.5% and 2.5% at 7 days, and 35% and 15% at 10 days p.i. for IC6452 and IC315022, respectively. The number of gRNA copies per infected mosquitoes was not significantly different between the two chimeric viruses in the three tissues within each time point (Fig 4B, S4 Table). Similar to what we observed for PR isolates, infection kinetic appeared faster for IC6452 and DENV gRNA copies did not vary between the two viruses.

Quantification of sfRNA copies and calculation of the sfRNA:gRNA ratio showed that the chimeric viruses recapitulated the two key observations made with the parental PR isolates. First, sfRNA:gRNA ratio was higher in salivary glands than in midgut and carcass (Fig 4C, S5 Table). The effect of virus was significant in our ANOVA model (S5 Table; P < 0.001) and, at 14 days which was the highest sfRNA level, there was at least a ten-fold higher sfRNA:gRNA ratio in salivary glands than in the two other tissues. Second, IC6452 had a higher sfRNA:gRNA ratio in salivary glands at 14 days p.i.. Additionally, we quantified gRNA, sfRNA and viral titers at 14 days p.i. in whole mosquitoes infected with IC6452 and IC315022. While the infection rate, DENV gRNA levels and viral titer were similar for the two chimeric viruses, the sfRNA:gRNA ratio was significantly higher after infection with IC6452 (S6 Fig). IC6452 and IC315022 recapitulated what we observed with PR6452 and PR315022, demonstrating that the 3’UTR sequence from the high EF isolates was responsible for higher sfRNA accumulation in salivary glands.

### The high epidemiological fitness 3’UTR increases production of virus progeny in salivary glands

**Fig 5:**
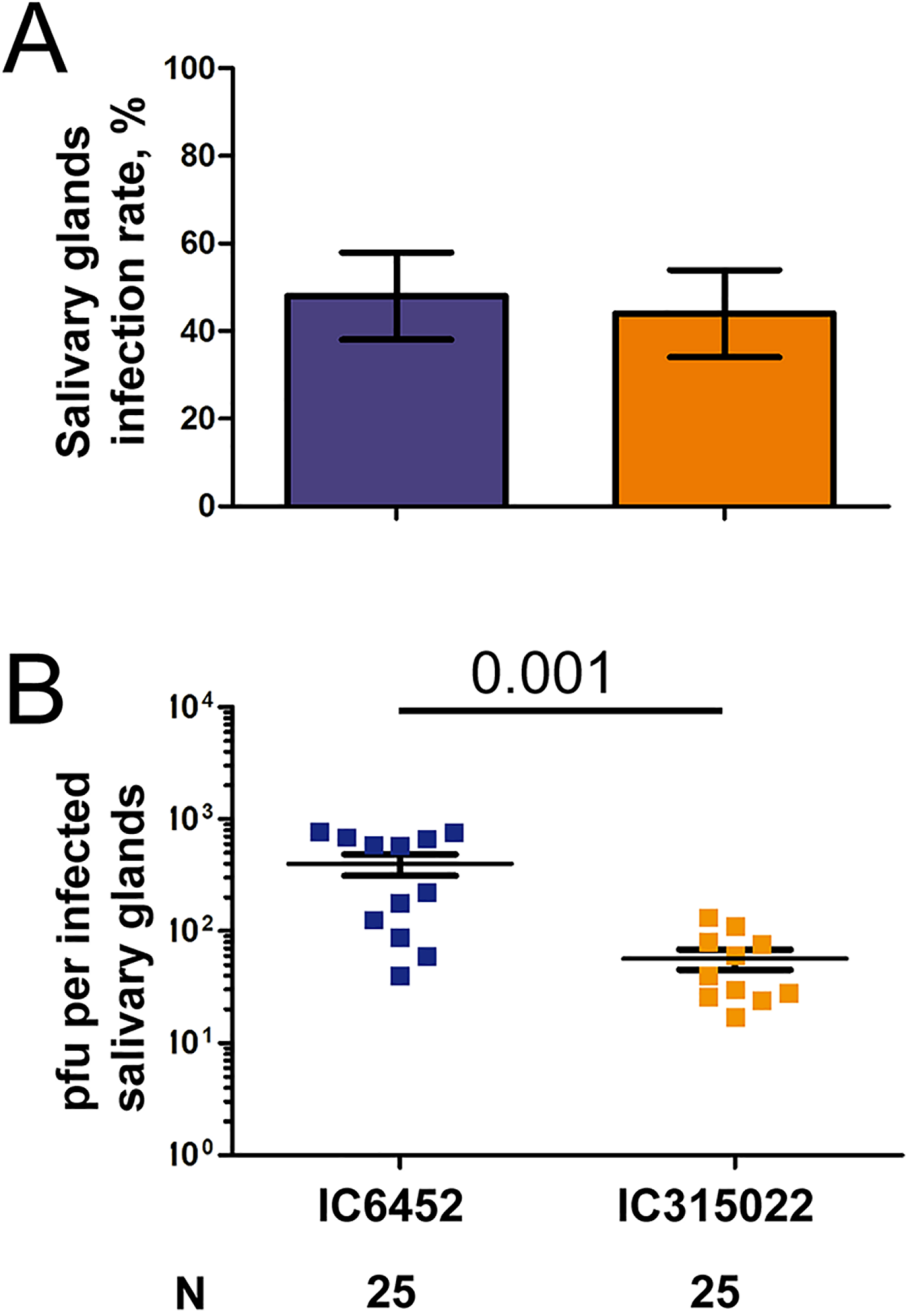
Virus titer in salivary glands is increased for mosquitoes infected with the chimeric virus containing the high epidemiological fitness 3’UTR sequence. Mosquitoes were orally infected with 10^6^ pfu / ml of the chimeric viruses, IC6452 or IC315022, containing the high or low EF 3’UTR sequence, respectively. At 14 days p.i., salivary glands were dissected and virus titer was quantified using plaque assay. (A) Infection rate in salivary glands. Bars represent percentages ± s.e. (B) plaque forming unit (pfu) per infected salivary glands. Only infected samples were represented, used to calculate the average and to perform t-test. Each point represents a pair of salivary glands. Bars show mean ± s.e.m. N, number of samples analyzed.

While the 3’UTR sequence did not influence gRNA level and the infection rate in salivary glands at the peak of sfRNA accumulation (14 days p.i.) when quantified using RT-qPCR (Fig 4A), we tested whether 3’UTR sequence from high or low EF altered the production of infectious particles in salivary glands. Mosquitoes were orally infected with the chimeric viruses IC6452 or IC315022, and progeny virus in salivary glands was tittered using a plaque forming assay at 14 days p.i., which corresponded to the peak of sfRNA production (Fig 4C). The infection rates of salivary glands were not different after infection with either of the chimeric viruses (Fig 5A), and were comparable to the ones calculated by detecting gRNA (Fig 4A). Importantly however, the virus titer in salivary glands was seven-fold higher and significantly different (p = 0.001) after infection with IC6452 (Fig 5B). Altogether, our results suggest that 3’UTR sequence from the high EF virus increases the production of infectious particles in salivary glands.

### The high epidemiological fitness 3’UTR increases infection rate of saliva

**Fig 6:**
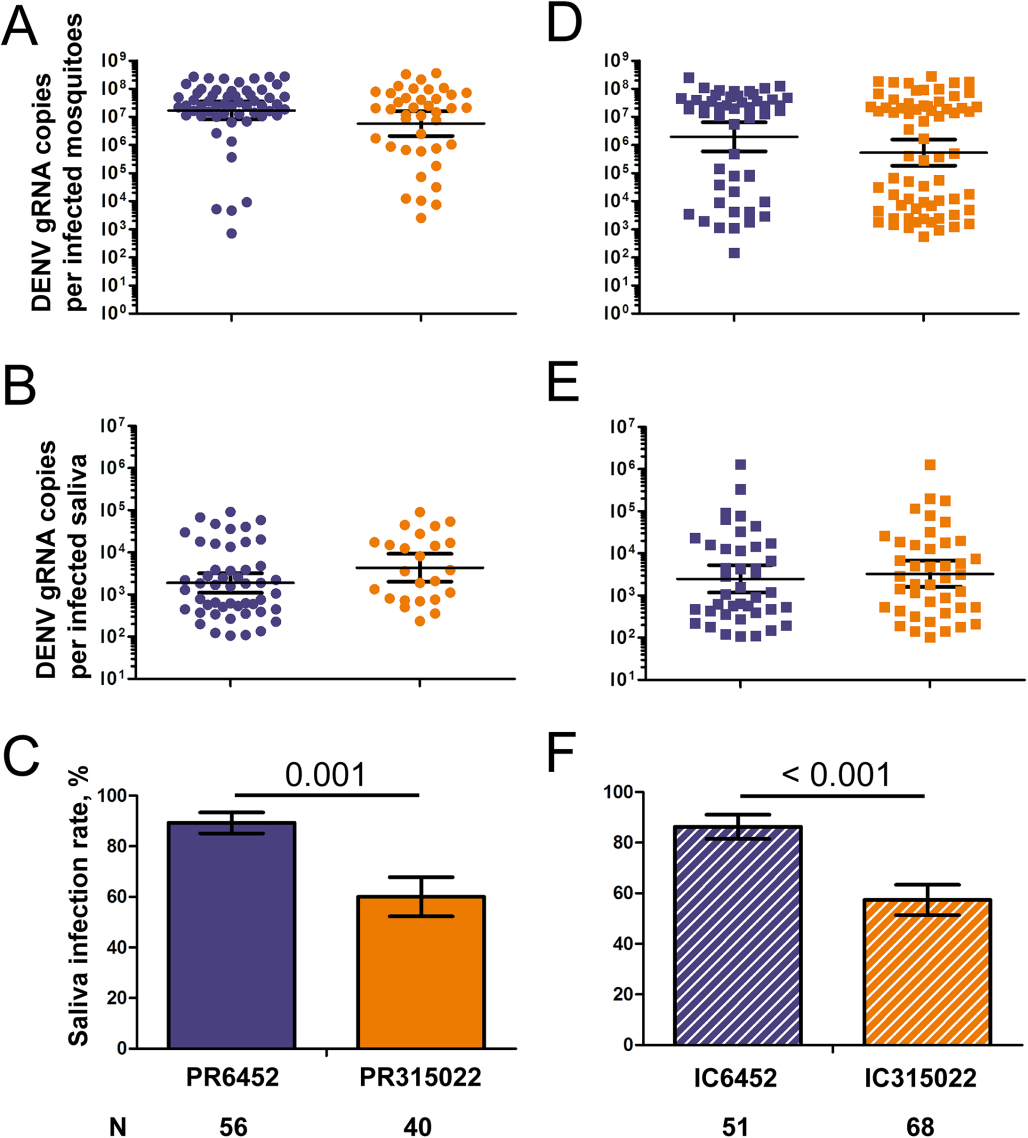
Infection rate of saliva is increased for mosquitoes infected with the viruses containing the high epidemiological fitness 3’UTR sequence. Mosquitoes were orally infected with 10^6^ pfu / ml of viruses, and let to expectorate in blood. DENV gRNA was quantified in mosquitoes and saliva. (A) DENV gRNA copies per infected mosquitoes, (B) DENV gRNA copies per infected saliva and (C) saliva infection rate 10 days p.i. with the high epidemiological fitness (EF) PR6452 or low EF PR315022. (D) DENV gRNA copies per infected mosquitoes, (E) DENV gRNA copies per infected saliva and (F) saliva infection rate 14 days p.i. with IC6452, containing the high EF 3’UTR, or IC315022, containing the low EF 3’UTR. N, number of infected mosquitoes that contained blood in their abdomen. (A, B, D, E) Bars show geometric mean ± 95% C.I. Only infected samples are represented. (C, F) Bars represent percentages ± s.e. Statistical differences between percentages were calculated using Z-test.

To test whether or not higher sfRNA:gRNA ratio and virus titer in salivary glands resulted in higher saliva infection rate (defined as the proportion of saliva containing DENV gRNA), we quantified DENV gRNA in saliva collected 10 days p.i. with PR6452 and PR315022. To avoid false negatives caused when a mosquito did not salivate, we collected saliva in blood and only analysed samples from infected mosquitoes with blood-containing abdomen. In our conditions, the rate of blood imbibing and the gRNA copies per infected whole mosquitoes were not different between the two isolates (Figs 6A and S7). While infected saliva contained the same quantity of DENV gRNA copies for both isolates (Fig 6B), the percentage of infected saliva was 28% (p = 0.001) higher for mosquitoes infected with PR6452 (Fig 6C). These data suggest that the more epidemiologically fit PR6452 is more likely to be transmitted than its less fit relative PR315022.

To determine the role of the 3’UTR in determining rate of saliva infection, we repeated the saliva quantification experiment with IC6452 and IC315022. Blood imbibing rate and DENV gRNA in whole mosquitoes were measured 14 days p.i., which corresponded to the highest observed sfRNA:gRNA ratio, and neither was different between the chimeric viruses (Figs S8 and 6D). As we observed above with the corresponding parental isolates, DENV gRNA in saliva was similar between chimeric viruses (Fig 6E), however, saliva infection rate was 29% (p < 0.001) higher after infection with IC6452 (Fig 6F). Altogether, these results indicate that the 3’UTR from the high EF PR6452 increases the sfRNA:gRNA ratio in salivary glands and the infection rate in saliva.

### 3’UTR sequences determine viral disruption of the immune response in salivary glands

**Fig 7:**
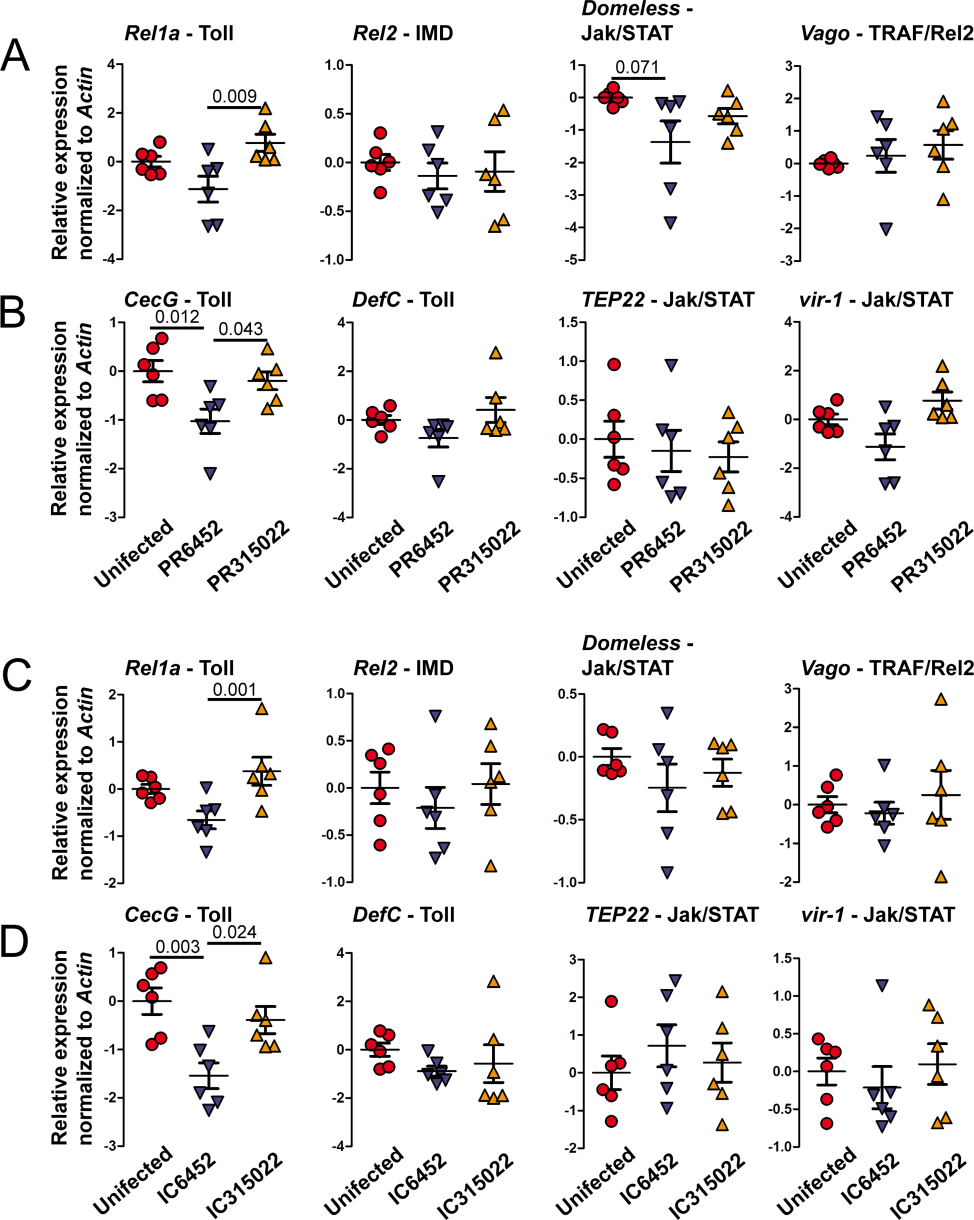
The Toll pathway is inhibited in salivary glands by PR6452 and the inhibition depends on 3’UTR sequences. Expression of (A) activators of Toll (*Rel1a*), IMD (*Rel2*), Jak/STAT (*Domeless*) and TRAF/Rel2 (*Vago*) immune pathways, and (B) of genes under the control of the Toll (*CecG* and *DefC*) or Jak/STAT (*TEP22* and *vir-1*) pathways at 10 days post-oral feeding with 10^6^ pfu / ml of PR6452, PR315022 or non-infectious blood (uninfected). Expression of (C) activators of Toll (*Rel1a*), IMD (*Rel2*), Jak/STAT (*Domeless*) and TRAF/Rel2 (*Vago*) immune pathways, and (D) of genes under the control of the Toll (*CecG* and *DefC*) or Jak/STAT (*TEP22* and *vir-1*) pathways at 14 days post-oral feeding with IC6452, IC315022 or non-infectious blood (blood). Gene expression was quantified by RT-qPCR and normalized to *actin*. Each point represents one sample grouping 10 salivary glands. Bars show mean ± s.e.m.

To test whether viruses with higher epidemiological fitness disrupt the mosquito immune response in salivary glands, we orally infected mosquitoes with PR6452, PR315022 or fed them with non-infectious blood. At 10 days post-oral feeding, which corresponded to the sfRNA:gRNA ratio peak in the salivary glands, we quantified gene expression for activators of Toll, IMD, Jak/STAT and Rel2/TRAF pathway in the salivary glands. We also tested these in midgut and carcass to validate the specificity of the immune disruption. We chose signalling activators that are up-regulated upon DENV infection in midgut or carcass [15, 16]; Rel1a as an activator of Toll, Rel2 of IMD, Domeless of Jak/STAT and Vago of TRAF/Rel2 [16, 41]. We first validated that DENV gRNA copies were similar between the two isolates for each tissue (S9 Fig). In salivary glands, *Rel1a* expression was downregulated 3.7 fold (p-value = 0.09) more by PR6452 than by PR315022, and *Domeless* was downregulated 2.6 fold (p-value = 0.071) more by PR6452 as compared to blood feeding while PR315022 did not alter *Domeless* expression (Fig 7A). Quantification of expression of the same immune markers in midgut and carcass showed that *Rel2* was significantly 1.87 times (p-value = 0.016) up-regulated in carcass infected with PR6452 as compared to PR315022 (S10A and S10B Fig). To confirm the inhibition of Toll and Jak/STAT pathways in salivary glands infected with PR6452, we quantified the expression of genes under the control of either of these pathways in the same samples. We chose to study Cecropin G (CecG) and Defensin C (Def C), two antimicrobial peptides which are positively regulated by Toll [42], and Thioester-containing protein 22 (TEP22), a component of the complement system in mosquitoes [43], and vir-1, which function is unknown, both positively regulated by Jak/STAT [11, 14, 15, 44]. In salivary glands, *CecG* RNA was significantly downregulated after infection with PR6452 as compared to both PR315022 (1.78 fold; p-value = 0.043) and non-infectious conditions (2.04 fold; p-value = 0.012) (Fig 7B). In midgut and carcass, however, only *TEP22* was significantly up-regulated in carcass upon PR6452 infection as compared to both PR315022 (2.05 times; p-value = 0.04) and non-infectious conditions (1.65 times; p-value = 0.04) (S10C and S10D Fig). These results strongly suggest that PR6452 partially disrupts the Toll pathway in salivary glands by inhibiting Rel1a expression.

To determine whether the 3’UTR was responsible for the differential immune disruption of the DENV2 isolates, we orally infected mosquitoes with the chimeric viruses IC6452 or IC315022, or fed them non-infectious blood. At 14 days post-oral feeding, which corresponded to the time where we observed the highest sfRNA:gRNA ratio, we quantified expression of the same immune pathway activators interrogated above. We first validated that DENV gRNA levels were similar between the two chimeric viruses in each tissue (S11 Fig). In salivary glands, while *Domeless* expression was 1.19 times lower after infection with IC6452 than after non-infectious blood feeding the difference was not significant (Fig 7C). Supportive of a role for the 3’UTR sequence in disrupting Toll pathway, *Rel1a* was downregulated 2.14 fold (p-value = 0.001) more by IC6452 than by IC315022. In midgut and carcass samples, only *Domeless* in carcass was 1.49 fold (p-value = 0.041) downregulated upon infection with IC6452 (S12A and S12B Fig). As we did for the parental viruses we quantified expression of genes under the control of Toll and Jak/STAT pathways. In salivary glands, *CecG* was downregulated 2.22 fold (p-value = 0.024) upon infection with IC6452 as compared to IC315022 (Fig 7D), whereas none of the genes were differentially expressed in midgut and carcass (S12C and S12D Fig). Altogether, these data demonstrate that 3’UTR sequences determine the preferential disruption of the Toll/Rel1a antiviral pathway in salivary glands infected with the high EF isolate PR6452.

## Discussion

By demonstrating a link between high EF 3’UTR DENV sequence on one hand, and increased sfRNA quantity, infectious particles in salivary glands, saliva infection rate, and immune disruption in the salivary glands on the other hand, we provide an explanation for how 3’UTR sequence evolution augments mosquito transmission.

Our study revealed an unprecedented tissue-specific pattern of sfRNA accumulation in DENV2-infected mosquitoes. The much higher sfRNA:gRNA ratio in salivary glands compared to other tissues suggests an important function for sfRNA in salivary glands, a critical organ for transmission. Furthermore, in salivary glands, but not in the midgut or carcass, we observed dramatically higher sfRNA levels with the high EF isolate when compared to the low EF one. We also observed an interesting temporal variation of sfRNA in salivary glands, where sfRNA peaked after 10 days for the parental isolates and 14 days for the chimeric viruses (Figs 3C and 4C). The time difference in sfRNA peaks may be attributed to the slower infection kinetic of infectious clones, as measured by the slower infection rate in the different tissues (Figs 3A and 4A), and may partially be caused by the limited virus genetic variation for the infectious clones [45]. Because sfRNA production comes at the expense of gRNA, which decrease can restrict replication, translation and packaging, sfRNA production might be optimized to match the transmission window and limit its associated cost. Accordingly, we observed that sfRNA peak levels occur after the extrinsic incubation period measured at the same temperature [9, 46]. It is intriguing to speculate that the high levels of sfRNA in salivary glands and the timing of its accumulation play important roles in transmission and perhaps infectivity of the virus in the mammalian host.

The tissue-specific overexpression of sfRNA raises several questions. We suspect that other tissues or cell types we did not capture in our analysis may also produce high levels of sfRNA. In fact, it should be noted that carcass samples consist of the material remaining left after dissection of salivary glands and midgut, and are mostly devoid of hemolymph fluids and loose tissues that can be infected [9]. This technical limitation impacts sfRNA quantification in dissected tissues as compared to that obtained with whole mosquitoes. In terms of a mechanism, sfRNA quantity is determined by production and degradation kinetics. Increased production could be the results of increased XRN1 activity; however, the lack of correlation between sfRNA:gRNA ratio and *XRN1* exonuclease gene expression does not support a determining role for XRN1 expression. These data do not refute possible increases in XRN1 protein or activity. In the same line of thought, increased production should concomitantly diminish gRNA levels, which we did not observe (Figs 3 and 4). Alternatively, binding of tissue-specific proteins to sfRNA could modulate susceptibility to XRN1 degradation or sfRNA stability. Salivary glands have different transcriptome as compared to carcass [47] and are thus expected to have a unique collection of protein capable of interacting with the sfRNA.

Using reverse genetics, we established a link between 3’UTR sequence and sfRNA quantity in whole mosquitoes and in salivary glands. Our group has previously demonstrated that single substitutions in a replicon are sufficient to alter sfRNA:gRNA ratio in human cells [30]. Three 3’UTR substitutions previously associated with high sfRNA:gRNA ratio in humans [30] are present in all our PR-2B isolates (S1 Fig). Two additional nucleotide substitutions in the PR-2B PR315022, a C to U transition at nucleotide 10,278 and a U to C transition at nucleotide 10,326, were sufficient to impair the gain in sfRNA:gRNA ratio in both human cells and mosquitoes. While we cannot definitively state which of the two substitution leads to low sfRNA levels for PR315022, we propose that U to C at 10,326 is the critical one. Using the recently solved structure of Zika virus sfRNA [48] as a model, we propose that this U to C transition disrupts the terminal loop and would weaken the pseudoknot pk1 and the long-range Watson-Crick base-pair between the bulged A in the long stem of xrRNA1 and the U at the base of the same structure (indicated for PR6452 in green in Fig 1H). These alterations are likely to make xrRNA1 much less resistant to XRN1 degradation reducing levels of full length sfRNA from PR315022. Indeed, the more dramatic reduction in sfRNA:gRNA ratio in PR315022 observed by RT-qPCR, which only measured full length sfRNA (Fig 1F), versus that seen in Northern blots, which detected all sfRNAs (Fig 1I), suggests that the longer sfRNA were likely the most affected by the PR315022 substitution. This is fully consistent with a recent study showing the existence of several species of sfRNA in DENV, with relative proportions influenced by point mutations in the 3’UTR [24]. The continuous unravelling of the plasticity of the nuclease-resistant structures indicates that multi-domain interactions are involved in determining sfRNA quantity. Correlated ratios of sfRNA:gRNA in human and mosquito further suggests a shared 3’UTR-based determinism of sfRNA:gRNA ratio, which could reduce dual-host constraints on 3’UTR evolution if sfRNA fitness advantage is also shared between the two hosts.

DENV infection induces an immune response in midgut through Toll and Jak/STAT signalling pathways [10, 15]. Artificial activation or repression of the Toll and Jak/STAT decreases or increases DENV replication, respectively, by regulating antiviral effectors [11, 12, 14, 15]. In DENV-infected salivary glands, Toll and IMD components are upregulated together with the antimicrobial Cecropin [13]. In another study, genes related to Toll pathway were found to be upregulated in uninfected salivary glands as compared to uninfected carcass and DENV infection was shown to induce several immune-related genes, although *Rel1a* was not regulated [47]. Interestingly, the different immune signalling pathways appear to co-regulate a set of identical genes as well as components from other immune pathways in mosquitoes [12, 15], suggesting cross-talks between signalling cascades. This is exemplified by the Rel2/TRAF pathway. Upon flavivirus infection in mosquito cells, TRAF is activated in a Dicer-2-dependent manner to induce Vago, which activates the Jak/STAT pathway and inhibits flavivirus replication [16]. In our study, salivary gland infection with the high EF isolate or the chimeric virus with the corresponding 3’UTR inhibited gene expression for the Toll component, *Rel1a*, and *Cecropin G* (*Cec G*), which is regulated by Toll [15]. Although the same gene expression pattern was observed at 10 and 14 days p.i. with the isolates and the chimeric viruses, respectively, modification of other immune pathways may occur earlier or later during salivary gland infection. We did not monitor gene expression of the RNAi components as they are not regulated upon DENV infection [15] and, hence, cannot be studied in our system. Nonetheless, sfRNA may alter the RNAi response as previously shown [25]. Inhibition of immune gene expression in DENV-infected cells was previously reported but no mechanism was provided [19]. Here, we observed that sfRNA quantity correlates with Toll pathway inhibition in salivary glands, strongly suggesting an immune-suppressor role for sfRNA in mosquitoes additional to its effects on RNAi. That one of the Toll-induced genes we tested, Defensin C, was not differentially regulated after infection by viruses containing the high EF 3’UTR is suggestive of a fine-tuned inhibition of the immune response. It should be noted that PR6452 not only produces more sfRNA than its less fit relative PR315022, but it also produces sfRNA(s) with different sequence (Fig 1H). The ability of different sfRNAs to bind innate immune regulators in human cells has been demonstrated [30, 49] and may function similarly in mosquitoes. Indeed, binding to innate immune regulators will be controlled by both sfRNA levels and sfRNA sequence variation. The evolution of sfRNA variants offers flaviviruses a flexible strategy to sample various anti-immune tactics [50].

Our study showed that the presence of a high EF 3’UTR increased the production of infectious particles but not accumulation of gRNA in salivary glands (Figs 4 and 5). Previous studies observed a decoupling of infectious particle production from gRNA replication in midgut during the course of a normal infection [9, 51]. In midgut, gRNA copies steadily increase throughout the infection, whereas infectious particles correlate with gRNA copies early during infection and decrease at later time points. Human immune response targets the difference steps of virus production, from replication, translation, packaging to virus egress [52]. While the impact of mosquito anti-DENV response remains to be characterized, our results show that inhibition of the Toll pathway correlates with higher number of infectious particles, which explains the increased probability that excreted saliva contains viruses. In conclusion, while outbreaks of DENV are influenced by multiple ecological and host factors [53, 54], viral characteristics are major contributors to epidemic transmission [55]. Here, we identified the 3’UTR as a genetic determinant of virus fitness in mosquitoes and report the first *in vivo* characterization of how dengue virus influences transmission through the production of sfRNA. Our study provides new mechanistic insights on the role of mosquito transmission in shaping the epidemiology of dengue, and, together with previous studies in mammals [30], emphasizes the importance of sfRNA evolution. Identification and characterization of viral determinants of mosquito transmission will help forecast emergence of DENV strains with higher epidemic potential.

## Materials and methods

### Mosquito rearing and maintenance

The *Ae*. *aegypti* colony was established in 2010 from eggs collected in Singapore. Adult mosquitoes were held in rearing cages (Bioquip) supplemented with 10% sucrose and fed pig’s blood (Primary Industries Pte Ltd) twice weekly to maintain the colony. After collection on wet papers, eggs were hatched in mili-Q water. Larvae were fed a mix of fish food (TetraMin fish flakes), yeast and liver powder (MP Biomedicals). The insectary was held at 28°C with 50% humidity on a 12:12h dark:light cycle.

### Virus isolates and propagation

Low-passage stocks of virus isolates were a gift from the Dengue Branch of the Centers for Disease Control and Prevention, Puerto Rico and were isolated in Eng Eong Ooi’s laboratory [30]. These isolates were generated after inoculating acute patient sera onto C6/36 cells (ATCC CRL-1660) as previously described [56]. These virus stocks were propagated in C6/36 cell, harvested 5 days post inoculation, aliquoted and stored at −80°C until use. All viral isolates included in this study were passaged 5–8 times. Viral titers were determined by plaque assay as previously reported [30].

### Construction of 3’UTR mutant viruses

An overlap cloning strategy was used to construct the full-length cDNA clones of DENV-2 (strain NGC) containing the 3’UTR mutations. Primers are detailed in S6 Table. Fragment A covering “MluI-NS5_partial_” was amplified with the primers 9127F and NS5-UTR-R using the DENV-2 NGC infectious clone as a template [57]. Fragment B was amplified from plasmids containing the desired 3’UTR with primers NS5-UTR-F and UTR-HDVr-R. Fragment C spanning “HDVr-to-XbaI unique site in pACYC” was amplified with primers UTR-HDVr-F and pACYC-11125-R using the DENV-2 NGC infectious clone as a template. Fragments A, B, and C were fused together to create cassette “MluI-NS5_partial_-3’UTR-HDVr-XbaI” by overlapping PCR with primers 9127F and pACYC-11125-R. The overlap PCR product was digested by MluI and XbaI and ligated into the full-length DENV-2 NGC infectious clone. All constructs were verified by DNA sequencing.

Full-length cDNA plasmids were linearized by XbaI and *in vitro* transcribed using a T7 mMessage mMachine kit (Ambion). The RNA transcripts (10 μg) were electroporated into BHK-21 cells following as described [58]. The transfected cells were seeded in a T-175 flask (8×10^6^ cells in 25 ml DMEM medium supplemented with 10% FBS) and incubated at 37^°^C for 24 h before culturing in DMEM medium with 2% FBS at 30^°^C. At every 24 h post-transfection, 200 μl culture fluids were collected and stored at -80^°^C. On day 5 post-transfection, all culture fluids were centrifuged at 4^°^C, 500 g for 5 min, aliquoted, and stored at -80^°^C. Viral titers were quantified by plaque assay using BHK-21 cell as described [30].

### Next generation sequencing of viruses

Total RNA from 500μl of the virus stocks was extracted using RNAzol RT (MRC). Library preparation was done using NEDNext Ultra Directional RNA Library Prep Kit (NEB). Next generation sequencing was run according to MiSeq Illumina paired-end protocol at the Duke-NUS Genome Biology Facility. Illumina sequences were trimmed using Trimmomatic [59]. Alignments were done with Geneious software v. 9.0.5. Sequences are available on GeneBank, accession SRX2617311-SRX2617315.

### Oral infection

Three to five day-old female mosquitoes were sugar-deprived for 24 h and subsequently offered a blood meal containing a 40% volume of washed erythrocytes from SPF pig’s blood (PWG Genetics), 5% of 100 mM ATP (Thermo Scientific), 5% human serum (Sigma) and 50% volume of virus in RPMI media (Gibco). The viral titer in the blood mix was 1x10^6^ pfu/ml and was validated by plaque assay as previously described [30]. Mosquitoes were exposed to the artificial blood meal for one hour using a Hemotek membrane feeder system (Discovery Workshops) with a porcine intestine membrane. Fully engorged females were selected and provided access to a 10% sugar solution in an incubation chamber with conditions similar to insect rearing. Mosquitoes were analyzed at different time points depending on the experiment.

### Quantification of gRNA and sfRNA

Single mosquitoes were homogenized in 350μl of TRK lysis buffer (E.Z.N.A. Total RNA kit I (OMEGA Bio-Tek)) using a bead Mill homogenizer (FastPrep-24, MP Biomedicals). Total RNA was extracted using E.Z.N.A. Total RNA kit I (OMEGA Bio-Tek) and eluted in 30μl of DEPC-treated water. Genomic RNA (gRNA) was quantified with a one-step RT-qPCR with iTaq Universal probe kit (Bio-Rad) and primers and probes targeting the DENV-2 Envelope [60]. Subgenomic flaviviral RNA (sfRNA) was quantified with a one-step RT-qPCR with iTaq Universal Sybr kit (Bio-Rad) using primers targeting the beginning of the sfRNA sequence [29]. The 25 μl reaction mix contained 1 μM of forward and reverse primer, 0.125 μM of probe for gRNA only and 4μl of RNA extract. Quantification was conducted on a CFX96 Touch Real-Time PCR Detection System (Bio-Rad). Thermal profile was 50°C for 10 min, 95°C for 1 min and 40 cycles of 95°C for 10 sec and 60°C for 15 sec.

An absolute standard curve was generated by amplifying fragments containing the qPCR targets (one fragment for each target) using forward primers tagged with a T7 promoter; for gRNA we used 5'-CAGGATAAGAGGTTCGTCTG-3' and 5'-TTGACTCTTGTTTATCCGCT-3', resulting in a 453bp fragment; for sfRNA we used 5'-AGAAGAGGAAGAGGCAGGA-3' and 5'-CATTGTTGCTGCGATTTGT-3', resulting in a 319bp fragment. The fragments were reverse transcribed using MegaScript T7 transcription kit (Ambion) and purified using E.Z.N.A. Total RNA kit I. The total amount of RNA was quantified using a Nanodrop (ThermoScientific) to estimate copy number. Ten times serial dilutions were made and used to generate absolute standard equations for gRNA and sfRNA (S4 Fig). In each subsequent RT-qPCR plate, quantification of four standard aliquot dilutions were used to adjust for threshold variation between plates.

Results from gRNA quantification were used to calculate the infection rate, which was the number of infected samples divided by the number of analyzed samples, and the average of gRNA copies per organ, which was calculated only from infected samples. Results from sfRNA and gRNA quantification were used to calculate the sfRNA:gRNA ratio by using only infected samples.

### Titration of virus in mosquitoes and salivary glands

Individual mosquitoes or pairs of salivary glands were homogenized in 500μl of RPMI, filtered through 0.22 μm filter (Sartorius) and titered using plaque assay with BHK-21(ATCC CCL-10) cells as previously described [30].

### Northern blot targeting 3’UTR

Total RNA was harvested from 50 mosquitoes 10 days p.i. with PR6452 or PR315022 using RNAzol RT (MRC). Northern Blot was conducted using NorthernMax kit (Ambion). We used the previously designed primers [29] to generate a biotin-16-dUTP (Roche) labelled dsDNA probe that complements 3’-UTR sequence (S1 Fig). Briefly, 32μg of total RNA mixed with loading dye was separated on a 1% agarose gel. The gel was then soaked in alkaline buffer (NaOH 0.01N and NaCl 3M) for 20 min and equilibrated 5 min in transfer buffer. Transfer to a nylon membrane (Biodyne B) was conducted by downward transfer as detailed in the NorthernMax kit protocol, with addition of a second bridge and buffer reservoir opposite to the first one, for 4h. After UV cross-linking, the membrane was subjected to two cycles of pre-hybridization with ULTRAhyb-oligo buffer (Ambion) at 42°C for 30min. 800ng of dsDNA labelled probe diluted in 10mM EDTA was denatured for 10 min at 95°C, immediately mixed with ULTRAhyb-oligo buffer and incubated overnight at 42°C on a rotating oven. Two low stringency wash at room temperature for 5 min and two high stringency wash at 50°C for 15 min were conducted. The membrane was blocked with 1% SDS Odyssey blocking buffer (TBS) (Li-cor) and stained at room temperature for 60 min with IRDYE 800cw streptavidin (Li-cor). Picture was taken with Odyssey CLx imaging system (Li-Cor) and image intensity, used to quantify blots, was measured with Image Studio Lite (Li-cor).

### Quantification of gene expression in different tissues

Salivary glands, midguts and carcasses from 10 orally infected mosquitoes were dissected and total RNA was extracted using E.Z.N.A. Total RNA kit I. Genomic DNA was removed using RapidOut DNA Removal kit (Thermo Scientific). Reverse transcription was performed with iScript cDNA Synthesis Kit (Bio-Rad) and gene expression was quantified using iTaq Universal SYBR Green Supermix (Bio-Rad) for *Rel1a*, *Rel2*, *Domeless*, *Vago*, *CecG*, *CecD*, *DefC*, *vir-1* and *TEP22* with primers detailed in S7 Table. *Actin* expression was used for normalization and quantified using primers detailed in S7 Table. Quantification was conducted on a CFX96 Touch Real-Time PCR Detection System (Bio-Rad). Thermal profile was 95°C for 1 min and 40 cycles of 95°C for 10 sec and 60°C for 15 sec. Six repeats were conducted.

### Quantification of virus genome in the saliva

Orally infected mosquitoes were cold-anesthetized and severed from their wings and legs. Their proboscis was inserted into a 10μl filter tip containing 10μl of SPF blood. Mosquitoes were allowed to expectorate for 30 min. Total RNA was extracted from the blood and the mosquitoes and DENV gRNA copies quantified as detailed above. The analysis was conducted only on mosquitoes that could be visually identified as having consumed blood and that were infected following RT-qPCR analysis. Infection rate of saliva was calculated by dividing the number of DENV-positive saliva over total number of saliva.

### Statistical analysis

The effects of virus on log-transformed DENV gRNA copies per infected mosquito, pfu per infected mosquitoes, sfRNA:gRNA ratio and gene expression in different tissues were analyzed using one-way ANOVAs. For the kinetic experiment, the influence of tissue, day of collection and virus strain on log-transformed DENV gRNA copies per infected mosquito and sfRNA:gRNA ratio were analysed using three-way ANOVA. The effects of virus and tissue on *XRN1* expression was tested using two-ANOVA. Post-hoc Tukey’s tests with Bonferroni adjustment were conducted when the effect was significant in the ANOVA test. Percentage differences were tested using a Z-test. In the saliva experiment, the effect of viruses on DENV gRNA copies per infected mosquitoes and per infected saliva were analyzed using Mann-Whitney’s non-parametric test. All tests were calculated using Systat 13.0 software (SYSTAT).

## Supporting information

S1 Fig Alignment of the 3’UTR sequences from all isolates used to infect mosquitoes (This figure relates to Fig 1). Nucleotide variations are highlighted. XRN1-resistant RNA structure (xrRNA) positions are shown. Positions for forward (qPCR For.) and reverse (qPCR Rev.) primers used in RT-qPCR, and for northern blot (NB) probe are shown.
(TIF)

S2 Fig Mosquito feeding rate on blood spiked with the same concentration of the different PR isolates (This figure relates to Fig 1). Mosquitoes were offered a blood meal spiked with virus. Blood engorged mosquitoes were selected under microscope and the feeding rate was determined over three biological repeats. Blood-feeding rate for (A) independent isolates and (B) the same isolates grouped according to epidemiological fitness (EF). Bars with a different letter were significantly different following Tukey’s test (A) or T-test (B). Bars show percentages ± s.e. N, number of mosquitoes that were offered a blood meal. NS, non-significant.
(TIF)

S3 Fig Mosquito survival post-oral infection with the same concentration of different PR isolates (This figure relates to Fig 1). Mosquitoes were offered a blood meal containing the same concentration of viruses. Engorged mosquitoes were then kept with sugar and water solutions until the day of observation for survival. Survival of mosquitoes at 10 days (A) for the different isolates and (B) the same isolates grouped by epidemiological fitness (EF) level, and (C) at 21 days. N, number of engorged mosquitoes. Bars with different letters are significantly different following a Z-test (A, C) or a t-test (B). Bars show percentages ± s.e. NS, non-significant.
(TIF)

S4 Fig **Standard curves for quantification of DENV gRNA (A) and sfRNA (B) (This figure relates to Fig 1).** DENV-2 RNAs that included the qPCR targets for DENV gRNA or sfRNA were generated *in vitro* by T7 RNA polymerase, their concentration was quantified using Nanodrop and used as 10 time serial dilutions for RT-qPCR. An equation was generated to quantify the absolute number of copies. Each Ct value was derived from three independent dilutions of the RNA stock.
(TIF)

S5 Fig *XRN1* mRNA variation in different tissues and after infection with PR6452 and PR315022. **(This figure relates to Fig 3).** Ten days after oral infection with either PR6452, PR315022 or non-infectious blood, salivary glands (SG), midguts and carcasses were dissected. (A) Log-2 *XRN1* mRNA expression normalized to the expression of *Actin*. (B) Log-2 *XRN1* mRNA expression normalized to the relative quantity of DENV gRNA copies. Six repeats with ten mosquitoes each were conducted. Each point represents one repeat and bars show mean ± s.e.m. Tables below the figures show results from a two-way ANOVA testing the impact of isolate and tissue on *XRN1* relative expression.
(TIF)

S6 Fig Higher ratio of sfRNA:gRNA in mosquitoes infected with a chimeric virus (IC6452) containing the 3’UTR from the high EF strain than in mosquitoes infected with a chimeric virus (IC315022) containing the 3’UTR from the low EF strain (This table relates to Fig 4). Mosquitoes were orally infected with the chimeric viruses containing either the 3’UTR of the high epidemiological fitness (EF) virus (IC6352) or the 3’UTR of the low EF virus (IC315022). At 14 days post-oral infection, (A) the gRNA, (B) ratio of sfRNA:gRNA and (C) the viral titer were measured in whole mosquitoes. Two different experiments were conducted to quantify the gRNA and the ratio of sfRNA:gRNA on one hand and the viral titer on the other hand. Infection rate was calculated for each experiment. N, number of mosquitoes analyzed.
(TIF)

S7 Fig Blood imbibing rate during saliva collection for PR6452- and PR315022-infected mosquitoes (This figure relates to Fig 6). At 10 days p.i. with PR6452 and PR315022, saliva was collected in blood. Blood imbibing rate was calculated after visual observation of the presence of blood in abdomen. Four repeats were conducted. Bars show percentages ± s.e. N, number of mosquitoes. NS, non-significant following Z-test.
(TIF)

S8 Fig Blood imbibing rate during saliva collection for IC6452- and IC315022-infected mosquitoes (This figure relates to Fig 6). At 14 days p.i. with IC6452 and IC315022, saliva was collected in blood. Blood imbibing rate was calculated after visual observation of the presence of blood in abdomen. Four repeats were conducted. Bars show percentages ± s.e. N, number of mosquitoes. NS, non-significant following Z-test.
(TIF)

S9 Fig Quantification of relative DENV gRNA copies in salivary glands, midguts and carcasses after infection with the isolates PR6452 and PR315022 (This figure relates to Fig 7). Mosquitoes were orally challenged with viruses and dissected into salivary glands (SG), midgut and carcass 10 days later. DENV gRNA copies was quantified using RT-qPCR and normalized to *actin* expression. Each point represents one sample containing specific tissue from 10 mosquitoes. Bars show mean ± s.e.m. Table shows results from a two-way ANOVA testing the effect of virus and tissue on relative DENV gRNA copies.
(TIF)

S10 Fig Expression of genes indicative of immune status in midguts and carcasses after infection with the PR6452 and PR315022 isolates (This figure relates to Fig 7). Mosquitoes were challenged with PR6452 or PR315022 or non-infectious blood (blood) and dissected into salivary glands (SG), midgut and carcass 10 days later. (A-B) Gene expression for activators of the Toll (*Rel1a*), IMD (*Rel2*), Jak/STAT (*Domeless*) and TRAF/Rel2 (*Vago*) immune pathways were quantified in (A) midgut and (B) carcass. (C-D) Expression of genes under the control of Toll (*CecG* and *DefC*) or Jak/STAT (*TEP22* and *vir-1*) pathways in (C) midgut and carcass (D). Gene expression was quantified by RT-qPCR and normalized to *actin*. Gene expression was calculated relative to gene expression in blood-fed salivary glands. Each point represents one sample grouping 10 salivary glands. Bars show mean ± s.e.m.
(TIF)

S11 Fig Quantification of relative DENV gRNA copies in salivary glands, midguts and carcasses after infection with the chimeric viruses IC6452 and IC315022 (This figure relates to Fig 7). Mosquitoes were orally challenged with viruses and dissected into salivary glands (SG), midgut and carcass 14 days later. DENV gRNA copies was quantified using RT-qPCR and normalized to *actin* expression. Each point represents one sample containing specific tissue from 10 mosquitoes. Bars show mean ± s.e.m. Table shows results from a two-way ANOVA testing the effect of virus and tissue on relative DENV gRNA copies.
(TIF)

S12 Fig Expression of genes indicative of immune activation in midguts and carcasses after infection with the chimeric viruses IC6452 and IC315022 (This figure relates to Fig 7). Mosquitoes were challenged with IC6452 or IC315022 or non-infectious blood (blood) and dissected into salivary glands (SG), midgut and carcass 14 days later. (A-B) Gene expression for activators of the Toll (*Rel1a*), IMD (*Rel2*), Jak/STAT (*Domeless*) and TRAF/Rel2 (*Vago*) immune pathways were quantified in (A) midgut and (B) carcass. (C-D) Expression of genes under the control of Toll (*CecG* and *DefC*) or Jak/STAT (*TEP22* and *vir-1*) pathways in (C) midgut and carcass (D). Gene expression was quantified by RT-qPCR and normalized to *actin*. Gene expression was calculated relative to gene expression in blood-fed salivary glands. Each point represents one sample grouping 10 salivary glands. Bars show mean ± s.e.m.
(TIF)

S1 Table Results of a three-way ANOVA testing the impact of the isolates, day of collection and tissue on the quantity of DENV gRNA copies per infected mosquitoes after infection with PR6452 or PR315022. (DOCX)

S2 Table Results of a three-way ANOVA testing the impact of the isolates, day of collection and tissue on the ratio of sfRNA:gRNA after infection with PR6452 or PR315022. (DOCX)

S3 Table Non-synonymous nucleotide differences between PR6452 and PR315022 genomes. (DOCX)

S4 Table Results of a three-way ANOVA testing the impact of virus, day of collection and tissue on the quantity of DENV gRNA copies per infected mosquitoes after infection with IC6452 or IC315022. (DOCX)

S5 Table Results of a three-way ANOVA testing the impact of virus, day of collection and tissue on the ratio of sfRNA:gRNA after infection with IC6452 or IC315022. (DOCX)

S6 Table Primer sequences used for construction of recombinant viruses. (DOCX)

S7 Table Primers for Real-Time qPCR. (DOCX)
