# Supplementary figures and images for: Dengue subgenomic flaviviral RNA disrupts immunity in mosquito salivary glands to increase virus transmission
Source: PLoS Pathog. 2017 Jul 28;13(7):e1006535. doi: 10.1371/journal.ppat.1006535 (PMC5555716; doi:10.1371/journal.ppat.1006535)

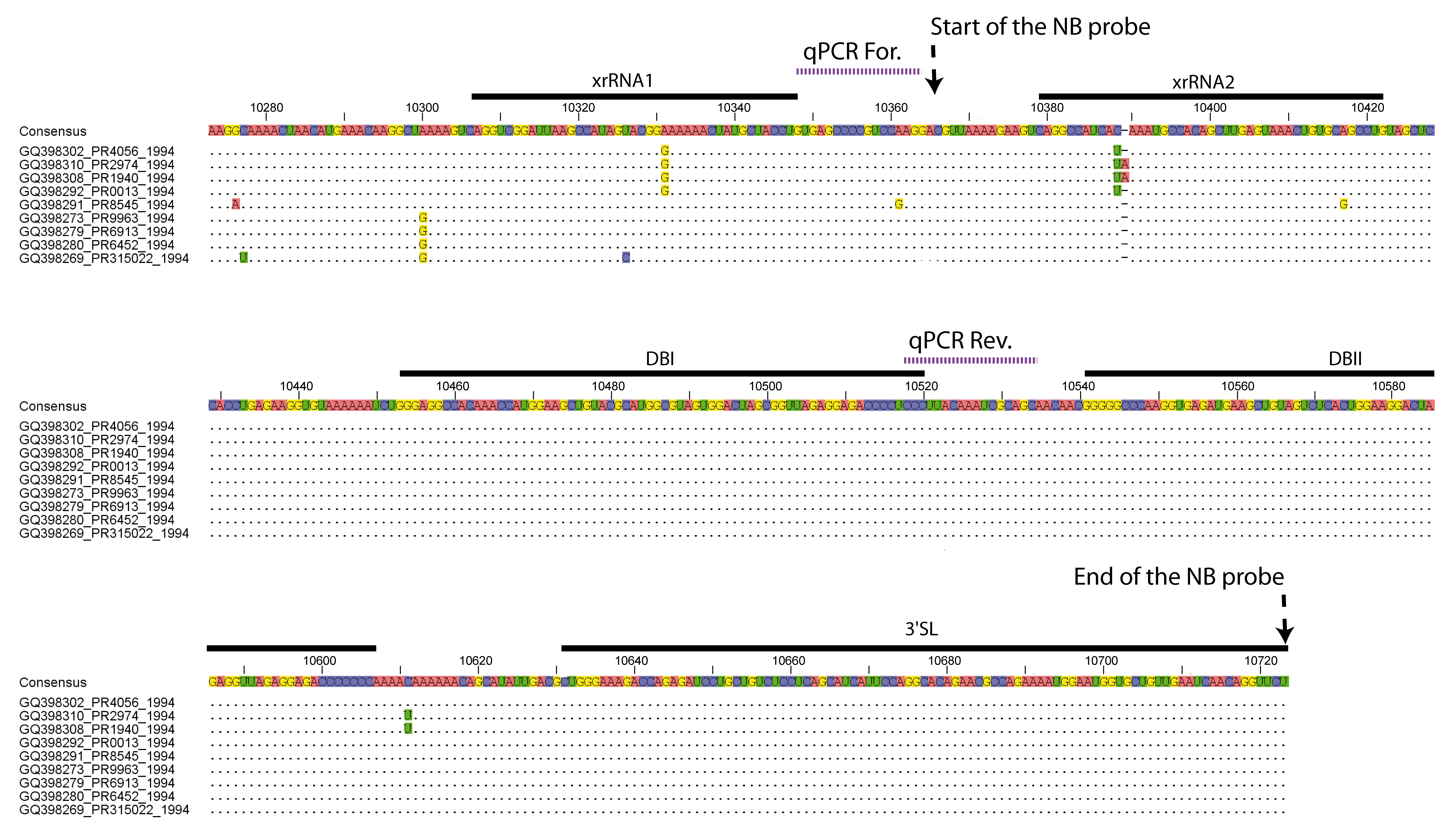

Supplement: S1 Fig — Nucleotide variations are highlighted. XRN1-resistant RNA structure (xrRNA) positions are shown. Positions for forward (qPCR For.) and reverse (qPCR Rev.) primers used in RT-qPCR, and for northern blot (NB) probe are shown. (TIF) [file ppat.1006535.s001.tif]

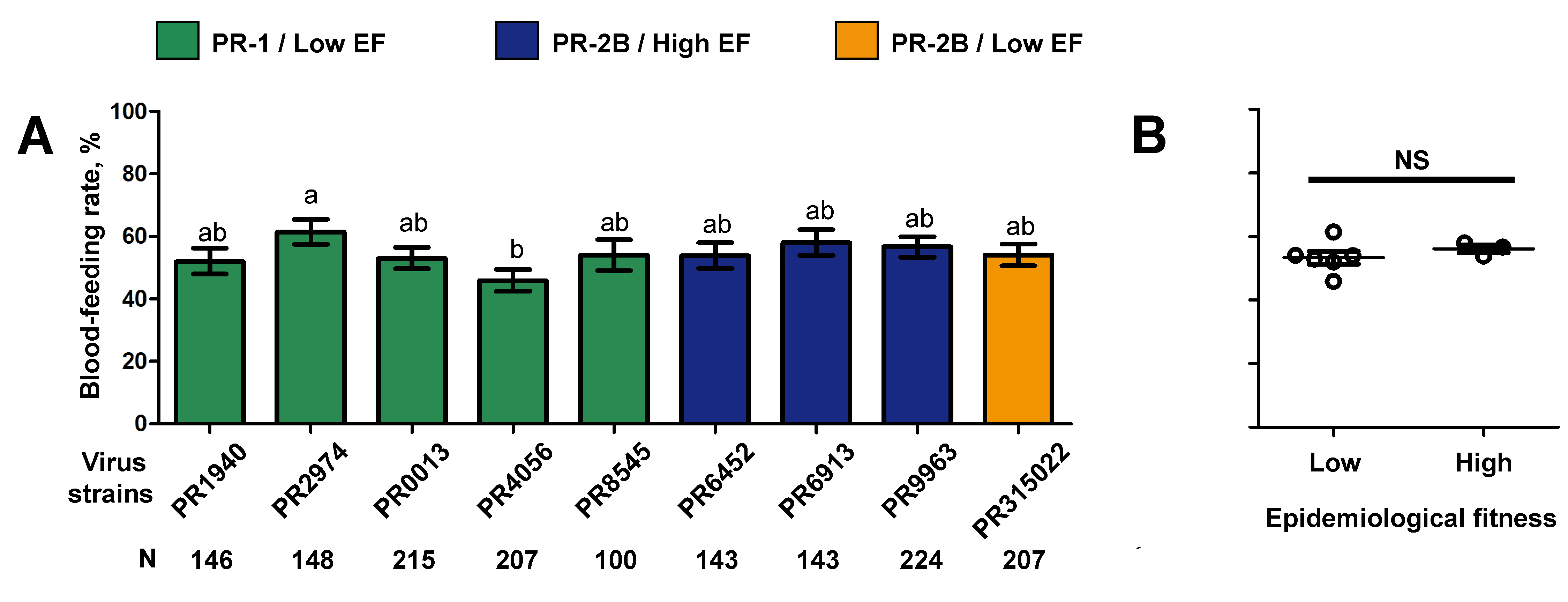

Supplement: S2 Fig — Mosquitoes were offered a blood meal spiked with virus. Blood engorged mosquitoes were selected under microscope and the feeding rate was determined over three biological repeats. Blood-feeding rate for (A) independent isolates and (B) the same isolates grouped according to epidemiological fitness (EF). Bars with a different letter were significantly different following Tukey’s test (A) or T-test (B). Bars show percentages ± s.e. N, number of mosquitoes that were offered a blood meal. NS, non-significant. (TIF) [file ppat.1006535.s002.tif]

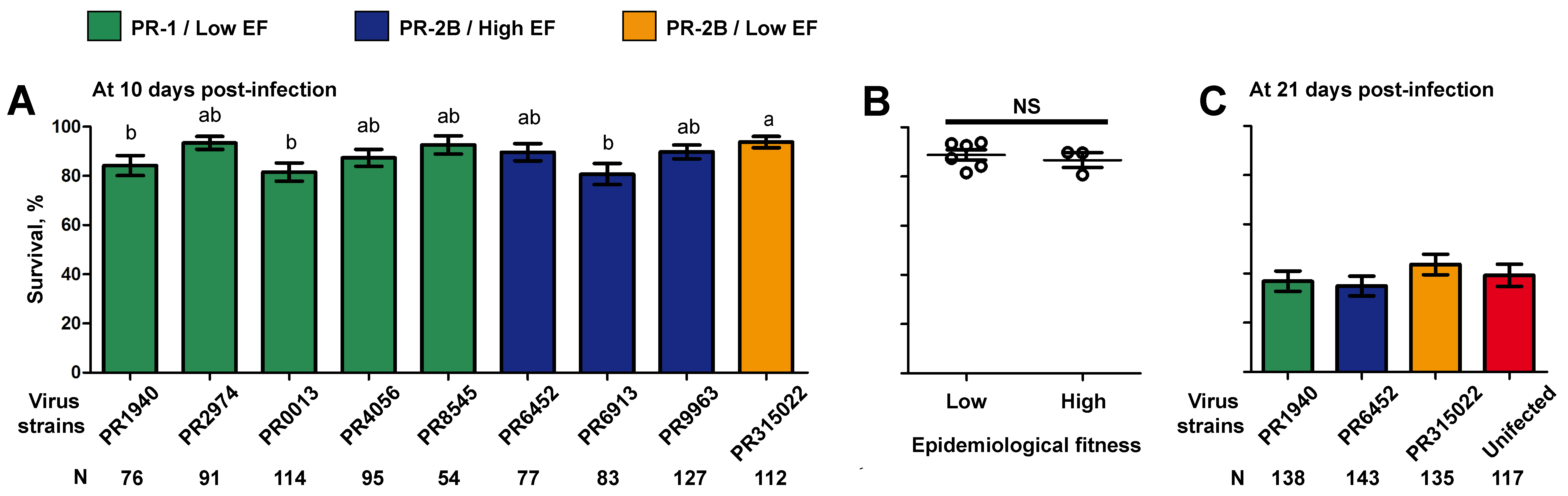

Supplement: S3 Fig — Mosquitoes were offered a blood meal containing the same concentration of viruses. Engorged mosquitoes were then kept with sugar and water solutions until the day of observation for survival. Survival of mosquitoes at 10 days (A) for the different isolates and (B) the same isolates grouped by epidemiological fitness (EF) level, and (C) at 21 days. N, number of engorged mosquitoes. Bars with different letters are significantly different following a Z-test (A, C) or a t-test (B). Bars show percentages ± s.e. NS, non-significant. (TIF) [file ppat.1006535.s003.tif]

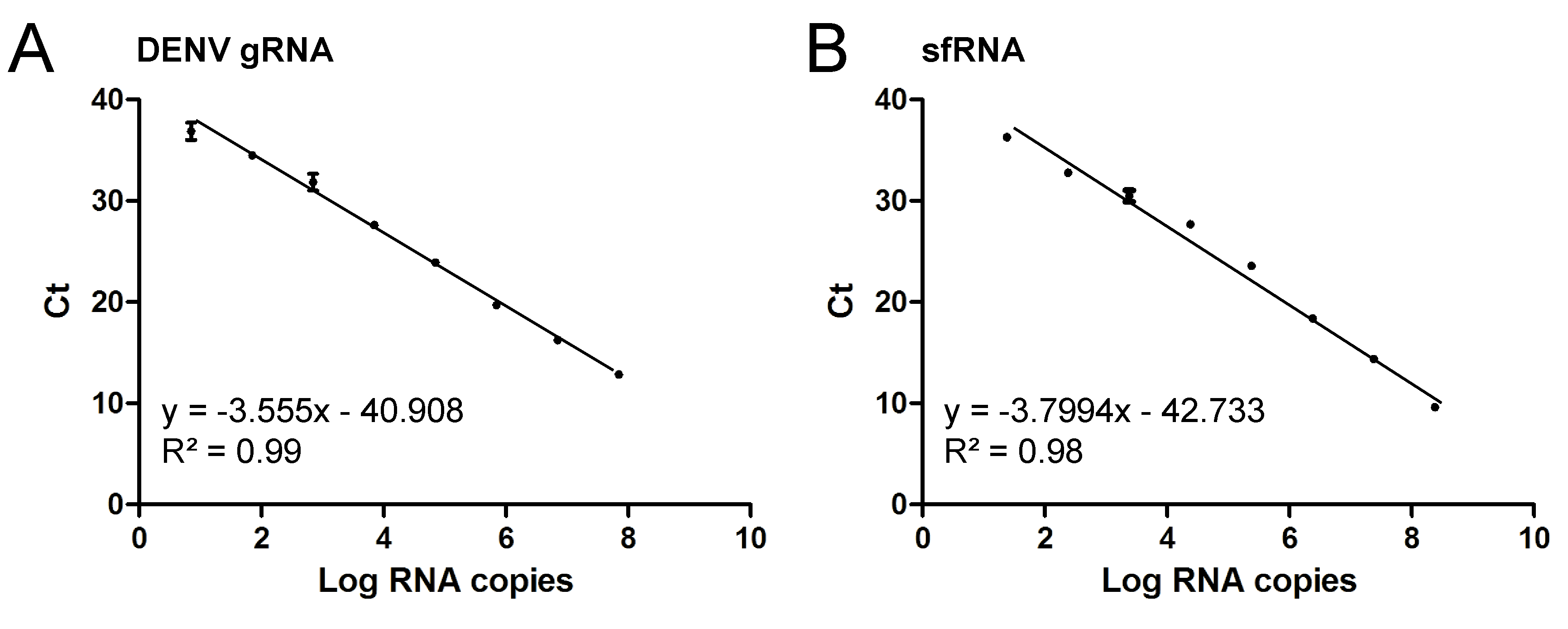

Supplement: S4 Fig — Standard curves for quantification of DENV gRNA (A) and sfRNA (B) (This figure relates to Fig 1). DENV-2 RNAs that included the qPCR targets for DENV gRNA or sfRNA were generated in vitro by T7 RNA polymerase, their concentration was quantified using Nanodrop and used as 10 time serial dilutions for RT-qPCR. An equation was generated to quantify the absolute number of copies. Each Ct value was derived from three independent dilutions of the RNA stock. (TIF) [file ppat.1006535.s004.tif]

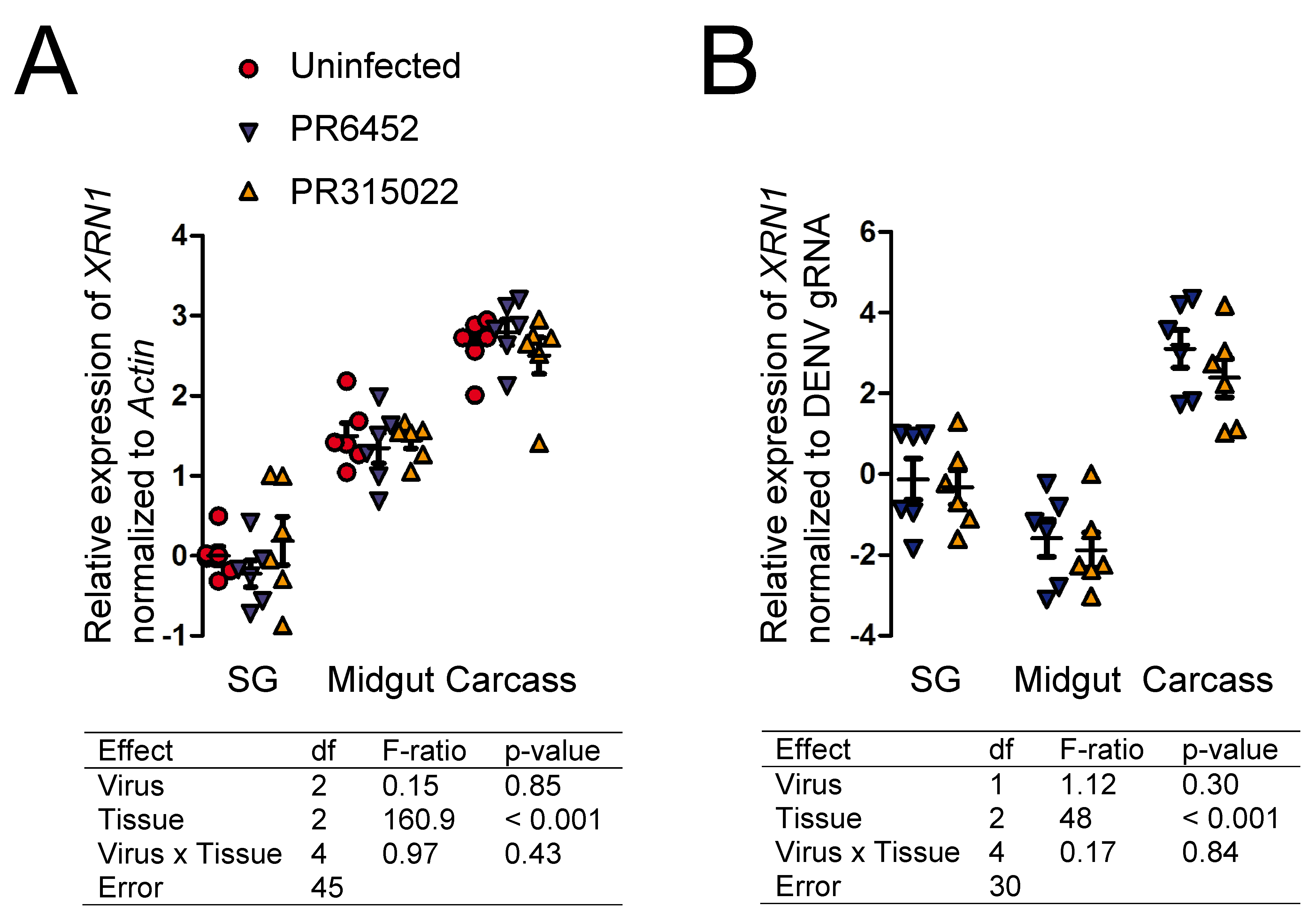

Supplement: S5 Fig — (This figure relates to Fig 3). Ten days after oral infection with either PR6452, PR315022 or non-infectious blood, salivary glands (SG), midguts and carcasses were dissected. (A) Log-2 XRN1 mRNA expression normalized to the expression of Actin. (B) Log-2 XRN1 mRNA expression normalized to the relative quantity of DENV gRNA copies. Six repeats with ten mosquitoes each were conducted. Each point represents one repeat and bars show mean ± s.e.m. Tables below the figures show results from a two-way ANOVA testing the impact of isolate and tissue on XRN1 relative expression. (TIF) [file ppat.1006535.s005.tif]

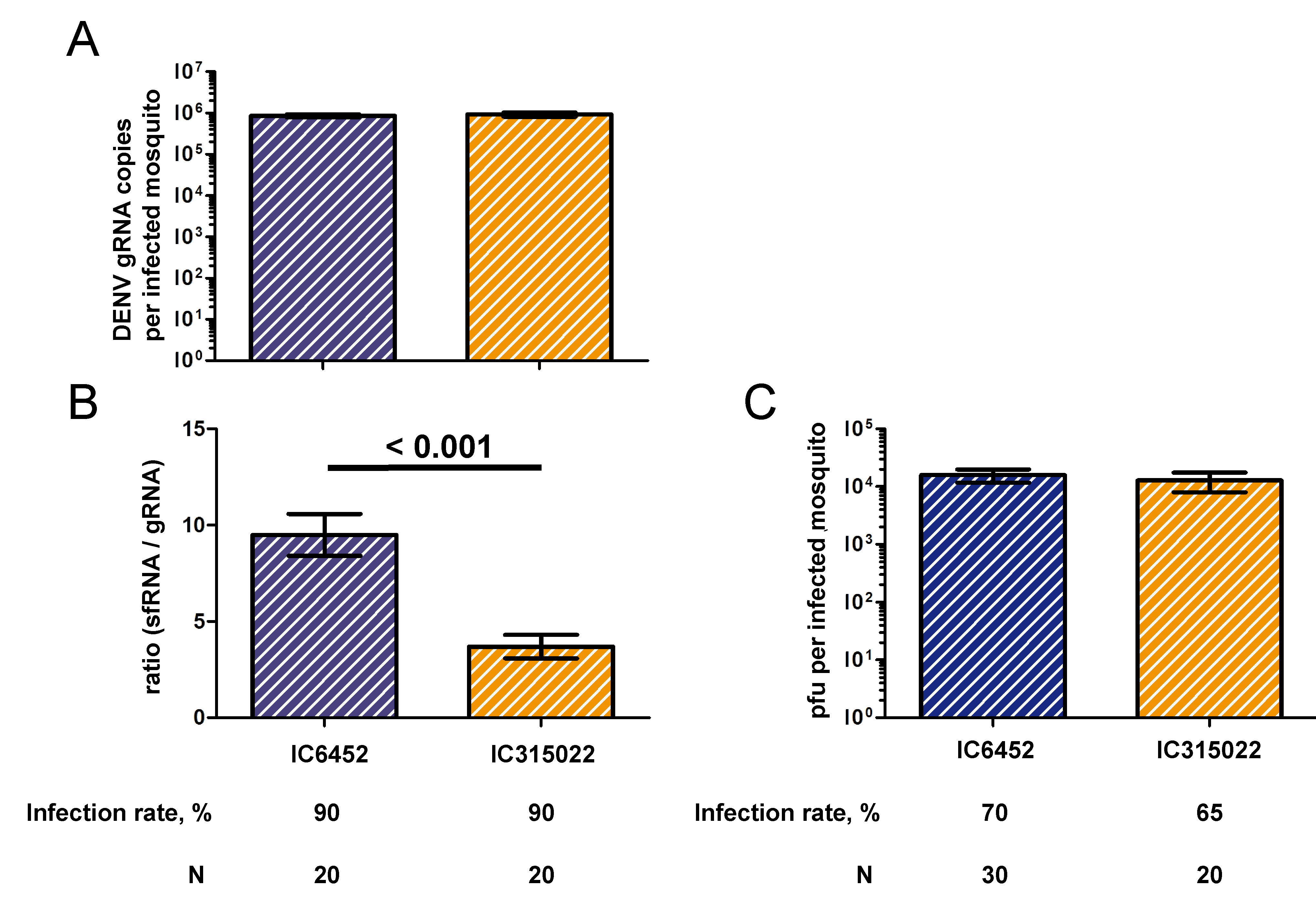

Supplement: S6 Fig — Mosquitoes were orally infected with the chimeric viruses containing either the 3’UTR of the high epidemiological fitness (EF) virus (IC6352) or the 3’UTR of the low EF virus (IC315022). At 14 days post-oral infection, (A) the gRNA, (B) ratio of sfRNA:gRNA and (C) the viral titer were measured in whole mosquitoes. Two different experiments were conducted to quantify the gRNA and the ratio of sfRNA:gRNA on one hand and the viral titer on the other hand. Infection rate was calculated for each experiment. N, number of mosquitoes analyzed. (TIF) [file ppat.1006535.s006.tif]

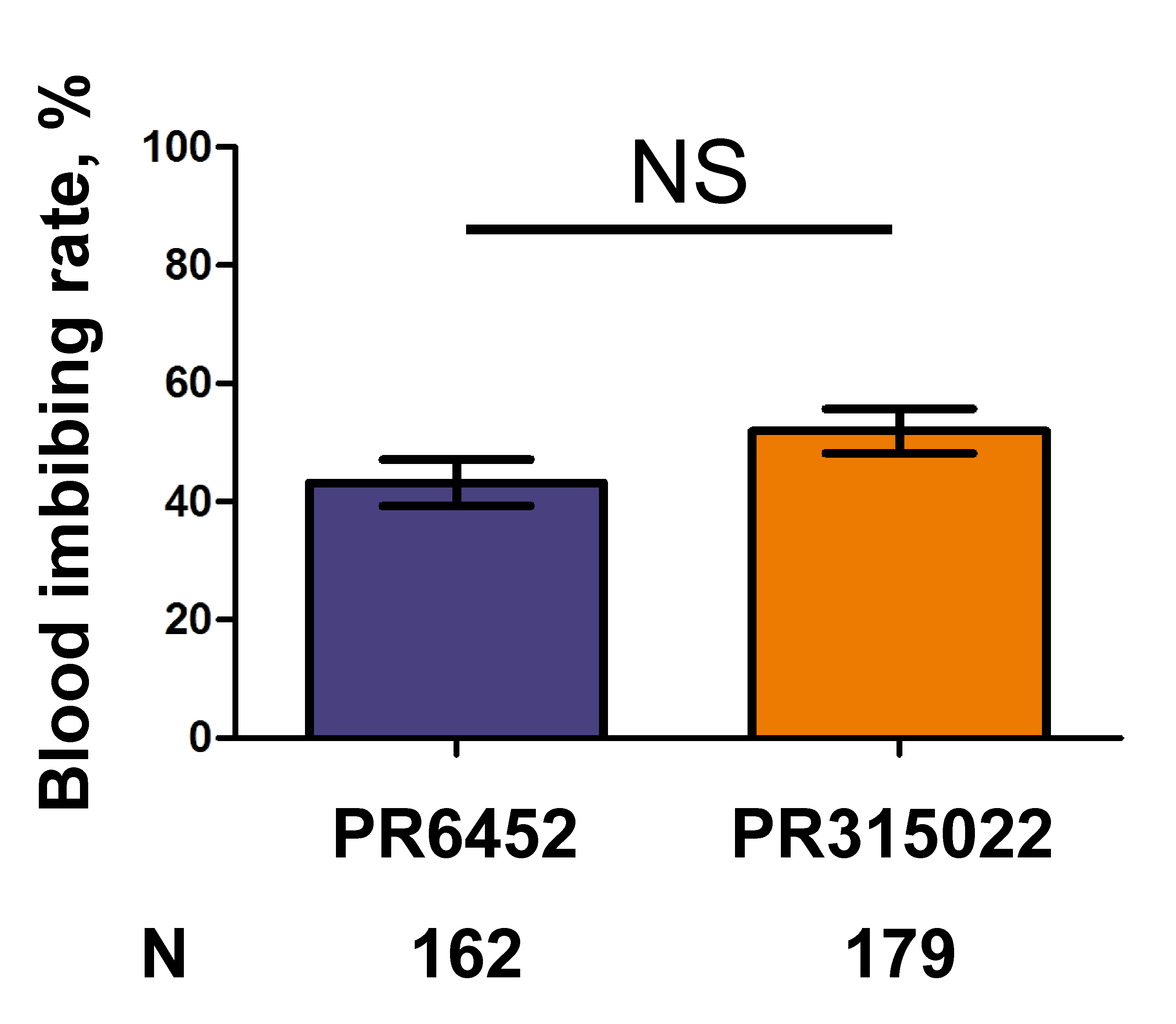

Supplement: S7 Fig — At 10 days p.i. with PR6452 and PR315022, saliva was collected in blood. Blood imbibing rate was calculated after visual observation of the presence of blood in abdomen. Four repeats were conducted. Bars show percentages ± s.e. N, number of mosquitoes. NS, non-significant following Z-test. (TIF) [file ppat.1006535.s007.tif]

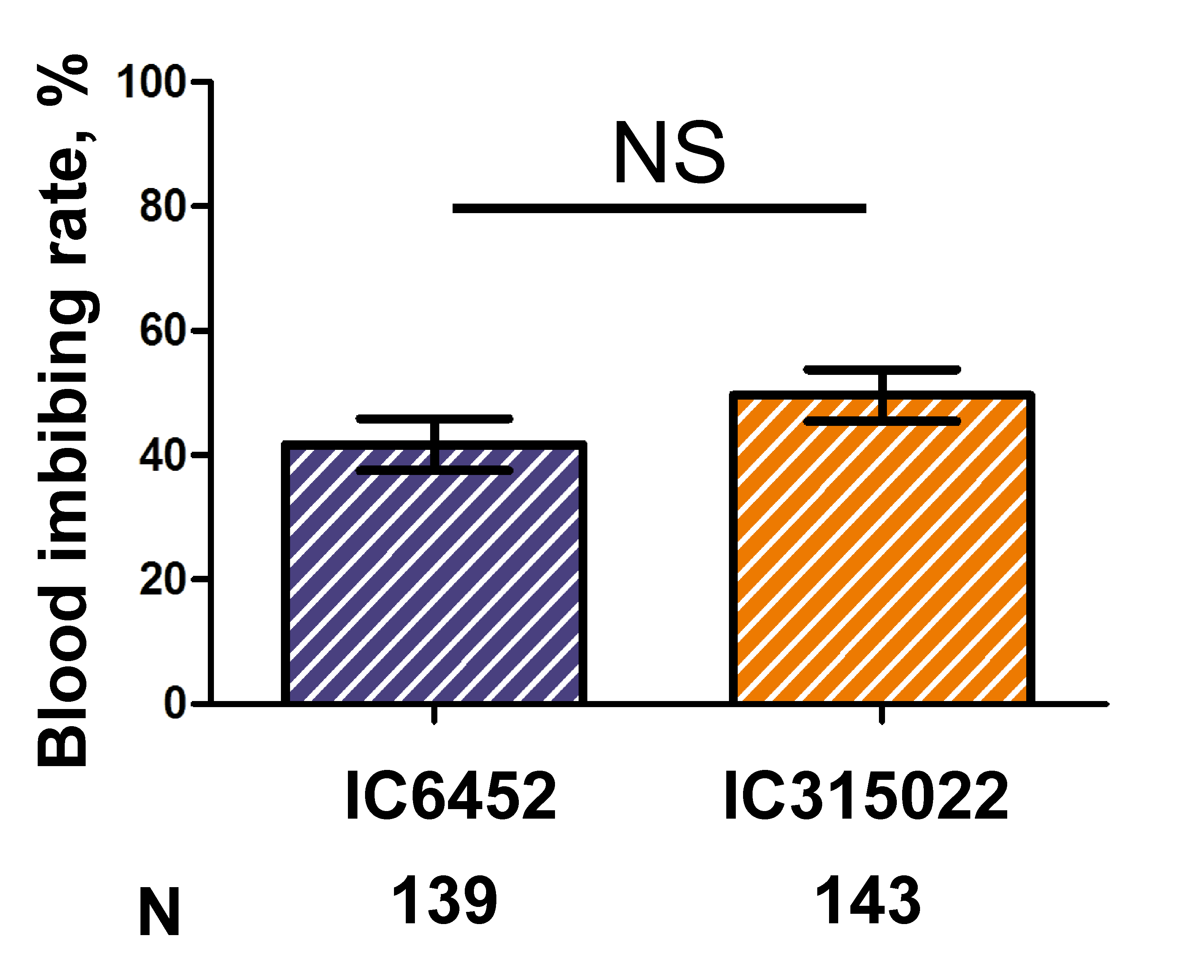

Supplement: S8 Fig — At 14 days p.i. with IC6452 and IC315022, saliva was collected in blood. Blood imbibing rate was calculated after visual observation of the presence of blood in abdomen. Four repeats were conducted. Bars show percentages ± s.e. N, number of mosquitoes. NS, non-significant following Z-test. (TIF) [file ppat.1006535.s008.tif]

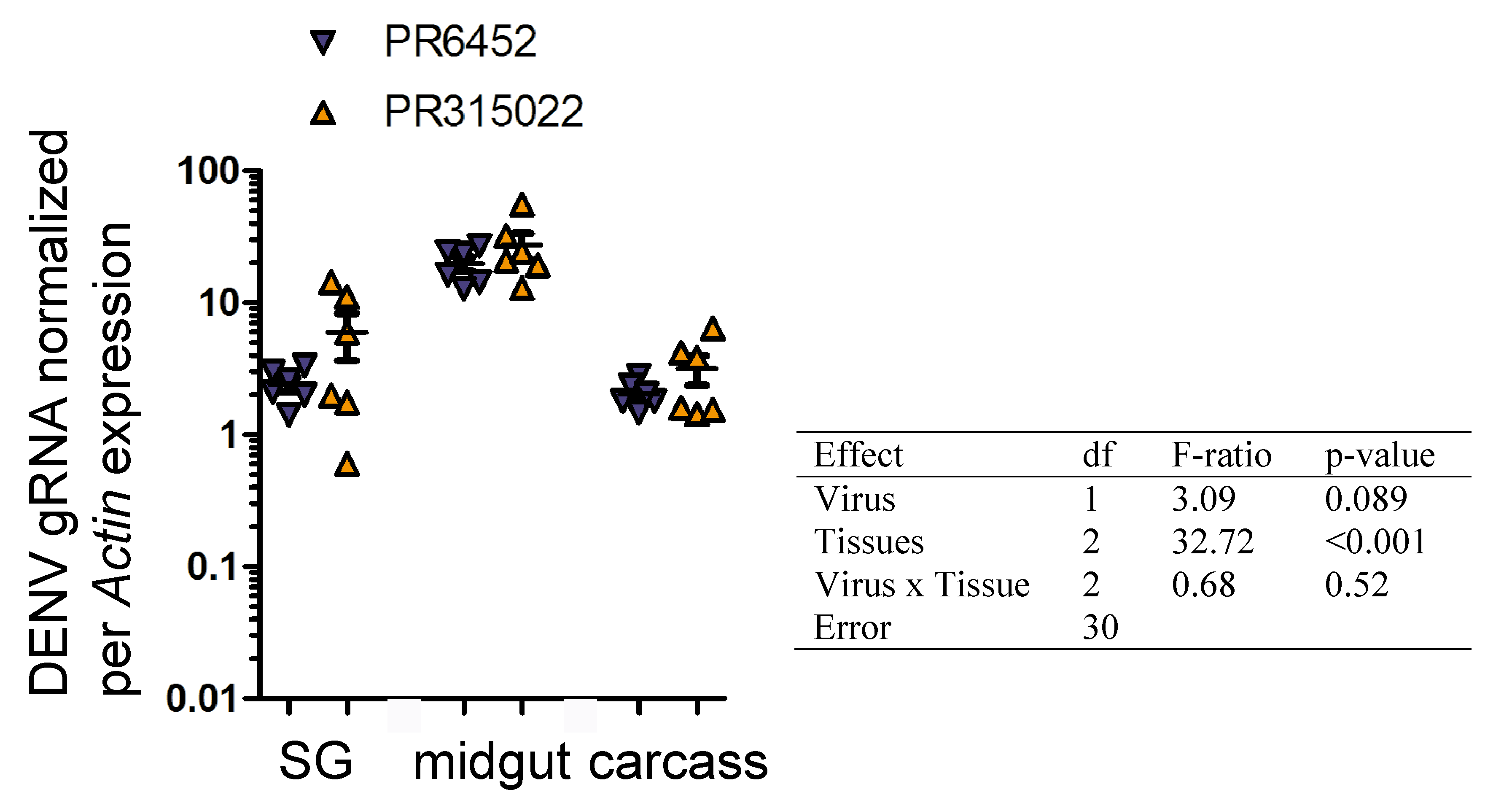

Supplement: S9 Fig — Mosquitoes were orally challenged with viruses and dissected into salivary glands (SG), midgut and carcass 10 days later. DENV gRNA copies was quantified using RT-qPCR and normalized to actin expression. Each point represents one sample containing specific tissue from 10 mosquitoes. Bars show mean ± s.e.m. Table shows results from a two-way ANOVA testing the effect of virus and tissue on relative DENV gRNA copies. (TIF) [file ppat.1006535.s009.tif]

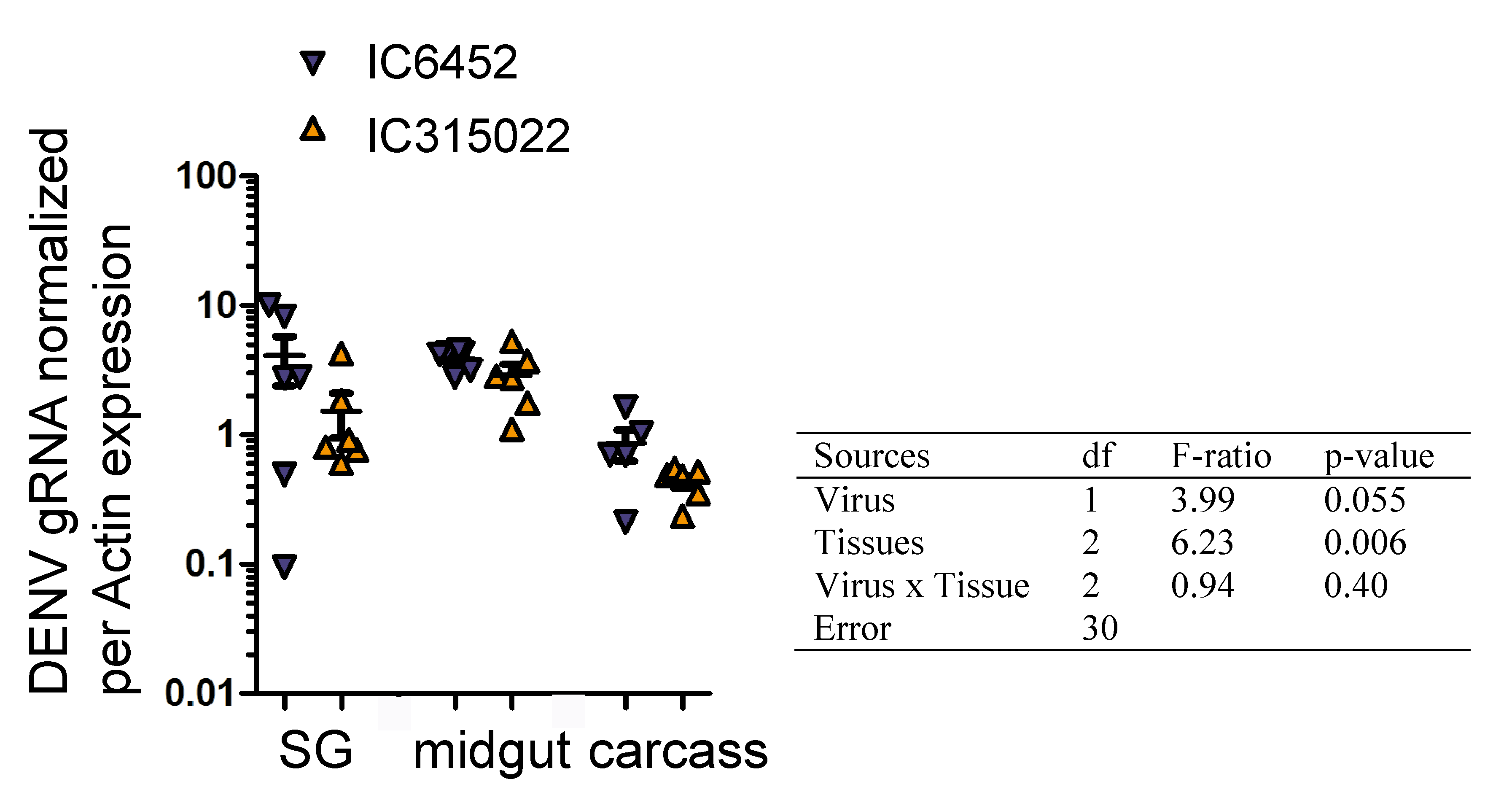

Supplement: S11 Fig — Mosquitoes were orally challenged with viruses and dissected into salivary glands (SG), midgut and carcass 14 days later. DENV gRNA copies was quantified using RT-qPCR and normalized to actin expression. Each point represents one sample containing specific tissue from 10 mosquitoes. Bars show mean ± s.e.m. Table shows results from a two-way ANOVA testing the effect of virus and tissue on relative DENV gRNA copies. (TIF) [file ppat.1006535.s011.tif]
