# Supplementary material for: Dengue subgenomic flaviviral RNA disrupts immunity in mosquito salivary glands to increase virus transmission
Source: PLoS Pathog. 2017 Jul 28;13(7):e1006535. doi: 10.1371/journal.ppat.1006535 (PMC5555716; doi:10.1371/journal.ppat.1006535)
Supplement: S1 Table — (DOCX) [file ppat.1006535.s013.docx]

**Table S1**. Results of a three-way ANOVA testing the impact of the isolates, day of collection and tissue on the quantity of DENV gRNA copies per infected mosquitoes after infection with PR6452 or PR315022.

| Effect | df | F-ratio | p-value |
| --- | --- | --- | --- |
| Isolates | 1 | 9.01 | 0.03 |
| Day of collection | 3 | 30.83 | < 0.001 |
| Tissue | 2 | 121.33 | < 0.001 |
| Isolates x Day of collection | 3 | 1.42 | 0.24 |
| Isolates x Tissue | 2 | 3.53 | 0.03 |
| Day of collection x Tissue | 6 | 18.61 | < 0.001 |
| Isolates x Day of collection x Tissue | 6 | 2.08 | 0.53 |
| Error | 660 |  |  |
