# Supplementary material for: Dengue subgenomic flaviviral RNA disrupts immunity in mosquito salivary glands to increase virus transmission
Source: PLoS Pathog. 2017 Jul 28;13(7):e1006535. doi: 10.1371/journal.ppat.1006535 (PMC5555716; doi:10.1371/journal.ppat.1006535)
Supplement: S2 Table — (DOCX) [file ppat.1006535.s014.docx]

**Table S2.** Results of a three-way ANOVA testing the impact of the isolates, day of collection and tissue on the ratio of sfRNA:gRNA after infection with PR6452 or PR315022.

| Effect | df | F-ratio | p-value |
| --- | --- | --- | --- |
| Isolates | 1 | 4 | 0.046 |
| Day of collection | 3 | 15.27 | < 0.001 |
| Tissue | 2 | 16.25 | < 0.001 |
| Isolates x Day of collection | 3 | 2.36 | 0.071 |
| Isolates x Tissue | 2 | 4.39 | 0.013 |
| Day of collection x Tissue | 6 | 8.67 | < 0.001 |
| Isolates x Day of collection x Tissue | 6 | 2.51 | 0.021 |
| Error | 660 |  |  |
