# Supplementary material for: Dengue subgenomic flaviviral RNA disrupts immunity in mosquito salivary glands to increase virus transmission
Source: PLoS Pathog. 2017 Jul 28;13(7):e1006535. doi: 10.1371/journal.ppat.1006535 (PMC5555716; doi:10.1371/journal.ppat.1006535)
Supplement: S3 Table — (DOCX) [file ppat.1006535.s015.docx]

**Table S3.** Non-synonymous nucleotide differences between PR6452 and PR315022 genomes.

| Nucleotide substitutions |  | PR6452 | | PR315022 | |
| --- | --- | --- | --- | --- | --- |
|  | Position | nt | AA | nt | AA |
| 5’UTR | 29 | A |  | G |  |
| Membrane | 679 | A | T | G | A |
| Envelop | 1544 | A | D | G | G |
| NS1 | 2660 | C | S | U | L |
|  | 2791 | C | L | U | F |
|  | 2911 | U | S | A | T |
|  | 3457 | G | V | A | I |
| NS2B | 4456 | G | V | C | L |
| NS3 | 4604 | G | R | A | K |
|  | 4612 | U | F | C | L |
|  | 4703 | G | R | A | K |
|  | 4879 | A | T | G | A |
|  | 4939 | G | V | A | I |
|  | 5078 | A | K | G | R |
|  | 5555 | A | N | G | S |
|  | 5716 | G | A | A | T |
|  | 5774 | G | R | A | K |
| NS4A | 6482 | U | V | C | A |
| NS4B | 6880 | A | I | G | A |
|  | 7348 | G | V | A | I |
| NS5 | 8375 | A | N | G | S |
|  | 9110 | A | K | G | R |
|  | 10241 | A | K | G | R |
| 3’UTR | 10277 | C |  | U |  |
|  | 10326 | U |  | C |  |
