# Supplementary material for: Dengue subgenomic flaviviral RNA disrupts immunity in mosquito salivary glands to increase virus transmission
Source: PLoS Pathog. 2017 Jul 28;13(7):e1006535. doi: 10.1371/journal.ppat.1006535 (PMC5555716; doi:10.1371/journal.ppat.1006535)
Supplement: S4 Table — (DOCX) [file ppat.1006535.s016.docx]

**Table S4**. Results of a three-way ANOVA testing the impact of virus, day of collection and tissue on the quantity of DENV gRNA copies per infected mosquitoes after infection with IC6452 or IC315022.

| Effect | df | F-ratio | p-value |
| --- | --- | --- | --- |
| Virus | 1 | 1.93 | 0.17 |
| Day of collection | 3 | 18.15 | < 0.001 |
| Tissue | 2 | 53.11 | < 0.001 |
| Virus x Day of collection | 3 | 1.30 | 0.275 |
| Virus x Tissue | 2 | 1.54 | 0.22 |
| Day of collection x Tissue | 6 | 3.52 | 0.002 |
| Virus x Day of collection x Tissue | 6 | 0.26 | 0.95 |
| Error | 531 |  |  |
