# Supplementary material for: Dengue subgenomic flaviviral RNA disrupts immunity in mosquito salivary glands to increase virus transmission
Source: PLoS Pathog. 2017 Jul 28;13(7):e1006535. doi: 10.1371/journal.ppat.1006535 (PMC5555716; doi:10.1371/journal.ppat.1006535)
Supplement: S5 Table — (DOCX) [file ppat.1006535.s017.docx]

**Table S5.** Results of a three-way ANOVA testing the impact of virus, day of collection and tissue on the ratio of sfRNA:gRNA after infection with IC6452 or IC315022.

| Effect | df | F-ratio | p-value |
| --- | --- | --- | --- |
| Virus | 1 | 3.86 | 0.050 |
| Day of collection | 3 | 6.26 | < 0.001 |
| Tissue | 2 | 8.57 | < 0.001 |
| Virus x Day of collection | 3 | 2.13 | 0.095 |
| Virus x Tissue | 2 | 2.96 | 0.053 |
| Day of collection x Tissue | 6 | 5.57 | < 0.001 |
| Virus x Day of collection x Tissue | 6 | 1.71 | 0.12 |
| Error | 531 |  |  |
