# Supplementary material for: Dengue subgenomic flaviviral RNA disrupts immunity in mosquito salivary glands to increase virus transmission
Source: PLoS Pathog. 2017 Jul 28;13(7):e1006535. doi: 10.1371/journal.ppat.1006535 (PMC5555716; doi:10.1371/journal.ppat.1006535)
Supplement: S6 Table — (DOCX) [file ppat.1006535.s018.docx]

**Table S6.** Primer sequences used for construction of recombinant viruses

| Primers^a^ | Sequence (5’-3’) |
| --- | --- |
| 9127F | AGA GAC GTG AGC AAG AAA GAG G |
| NS5-UTR-F | TCC ATG AAA AGA TTC AGA AAA GAA GAG GAA GAG GCA GG |
| NS5-UTR-R | CCT GCC TCT TCC TCT TCT TTT CTG AAT CTT TTC ATG GA |
| UTR-HDVr-F | TGT TGA ATC AAC AGG TTC TGG GTC GGC ATG GCA TCT CC |
| UTR-HDVr-R | GGA GAT GCC ATG CCG ACC CAG AAC CTG TTG ATT CAA CA |
| pACYC-11125-R | GGT GAG TAA CCA TGC ATC ATC AGG |

^a^The primers are named after the nucleotide position of viral sequence and polarity. F, forward primer representing viral genome sense; R, reverse primer representing complementary sense. Nucleotide numbering is based on DENV-2 strain NGC (GenBank number AF038403).
