# Supplementary material for: Dengue subgenomic flaviviral RNA disrupts immunity in mosquito salivary glands to increase virus transmission
Source: PLoS Pathog. 2017 Jul 28;13(7):e1006535. doi: 10.1371/journal.ppat.1006535 (PMC5555716; doi:10.1371/journal.ppat.1006535)
Supplement: S7 Table — (DOCX) [file ppat.1006535.s019.docx]

**Table S7.** Primers for Real-Time qPCR

| Gene name | Gene code | Forward primer | Reverse primer |
| --- | --- | --- | --- |
| *Rel1a* | AAEL007696 | ACCTTCAGCAACCTGGGCAT | GAGATCGATGGTGGCAGGCT |
| *Rel2* | AAEL007624 | TACGAGCTCCTTCAACATGC | AGGTCTGCAGTTGACCCTCT |
| *Domeless* | AAEL012471 | AAACGGTGGCAAAATGAACT | CATACAGCCGGCTTTCTTCT |
| *Vago* | AAEL000165 | ATTTCTCTTCATCGGGATCG | CTGCTTGATCGCAGTAGCAT |
| *Cecropin G (CecG)* | AAEL015515 | CGTGGCTGTTCTTCTCCTG | TCTTTCCCAGCTTCTTGAGG |
| *Defensin C (DefC)* | AAEL003832 | GTGGGTTCGGTGTAGGAGAT | CAATTTCGACAAACGCAAAC |
| *Vir-1* | AAEL000718 | GCCAAAGTCCGGTATTCTTC | TTCACGAGATCGTCAAGGTAA |
| *TEP22* | AAEL000087 | GCGGGACTGATTGTACCTTT | GAGGATATCGCCTGGTTTGT |
| *Actin* | AAEL011197 | GAACACCCAGTCCTGCTGACA | TGCGTCATCTTCTCACGGTTAG |
